# Common Skin Diseases and Metabolic Syndrome: A Proinflammatory Chemokine Perspective

**DOI:** 10.3390/metabo16040253

**Published:** 2026-04-10

**Authors:** Mateusz Matwiejuk, Hanna Myśliwiec, Agnieszka Mikłosz, Adrian Chabowski, Iwona Flisiak

**Affiliations:** 1 Department of Dermatology and Venerology, Medical University of Bialystok, 15-540 Bialystok, Poland; 2 Department of Physiology, Medical University of Bialystok, 15-222 Bialystok, Poland

**Keywords:** CCL5/RANTES, CXCL12/SDF-1a, CCL7/MCP-3, CCL2/MCP-1, CXCL1/GROa, eotaxin, skin, skin diseases, psoriasis, metabolic syndrome

## Abstract

Skin diseases frequently coexist with other disorders, such as metabolic syndrome, diabetes mellitus, depression, psoriatic arthritis, and cardiovascular disease. Altered levels of distinct chemokines, like CCL5/RANTES, CXCL12/SDF-1a, CCL7/MCP-3, CCL2/MCP-1, CXCL1/GROa, and the eotaxin family, contribute to the development and/or exacerbation of inflammation, which is a common feature of numerous skin diseases as well as metabolic syndrome. The pathological and molecular connections between chronic inflammatory skin diseases and metabolic syndrome are increasingly recognized as being driven by shared inflammatory pathways, oxidative stress, and adipokine dysregulation. While systemic inflammation acts as a common thread, the precise mechanisms for some conditions remain partially understood. Nevertheless, the exact pathological and molecular connections between skin diseases (i.e., psoriasis, atopic dermatitis, pemphigus vulgaris, acute and chronic spontaneous urticaria, bullous pemphigoid, squamous cell carcinoma, alopecia areata, systemic sclerosis, discoid lupus erythematosus, diffuse large B-cell lymphoma) and metabolic syndrome are not yet fully understood. This narrative review summarizes the robust association between various chronic inflammatory skin diseases and metabolic syndrome in the context of pro-inflammatory chemokines.

## 1. Introduction

This narrative review focuses on the crucial role of chemokines in the pathogenesis of several common skin diseases, including psoriasis, atopic dermatitis, systemic sclerosis, squamous cell carcinoma, alopecia, autoimmune bullous diseases, discoid lupus erythematosus, ulcers, and diffuse large B-cell lymphomas. Psoriasis is a common systemic inflammatory disease that affects approximately 1.12% of the global population [1]. It is estimated that around 55.8 million adults worldwide have psoriasis [1]. Psoriasis is mainly characterized by erythrosquamous plaques and varying degrees of pruritus [2]. Different chemokines cause hypertrophy of endothelial cells and fibroblasts, contributing to the development of the inflammatory process involved in many skin diseases. Chemokines are a family of small, inducible signaling proteins that mediate cell trafficking and migration (chemotaxis), particularly of leukocytes, in response to inflammatory stimuli (like IL-1, IFN-γ, and TNF-α). They are categorized into four subfamilies—CXC, CC, C, and CX3C—based on the structural arrangement of conserved N-terminal cysteine (C) residues [3]. Chemokines play a key role in the regulation of physiological mechanisms and are also involved in the pathogenesis of various diseases, such as psoriasis [3]. For instance, they contribute to the activation and recruitment of T lymphocytes, neutrophils, and macrophages to inflammatory sites. Skin disorders may coexist with metabolic abnormalities, including elevated blood sugar levels, hypertension, large waist, and dyslipidemia, components of metabolic syndrome (MetS) [4,5,6]. Chronic inflammation, driven largely by the dysregulation of the IL-23/Th-17 immune signaling pathway, serves as a key pathogenic link between inflammatory skin diseases—particularly psoriasis—and metabolic syndrome (MetS). While psoriasis is characterized by skin lesions, it is now considered a systemic disease where IL-23 sustains Th17 cells, leading to the production of IL-17, IL-22, and TNF-α, which in turn drive skin inflammation and promote systemic metabolic dysregulation [7]. Bimekizumab, a dual inhibitor of IL-17A and IL-17F, shows high efficacy and a favorable safety profile in obese patients with moderate-to-severe plaque psoriasis, making it a strong therapeutic option for this population. Real-world evidence demonstrates that obese patients achieve high rates of PASI 90/100, often exceeding the performance of other anti-IL-17 and anti-IL-23 agents [8,9].

Furthermore, administration of anti-IL-17A monoclonal antibodies, such as ixekizumab and secukinumab, improved hyperglycemia in patients with psoriasis and decreased fasting glucose levels in imiquimod-treated mice [10]. A better understanding of potential common pathways connecting metabolic syndrome and related skin diseases is of great importance for developing new therapeutic strategies. In this review, we outline the potential association between metabolic syndrome and skin diseases, including psoriasis, atopic dermatitis, pemphigus vulgaris, acute and chronic spontaneous urticaria, bullous pemphigoid, squamous cell carcinoma, alopecia areata, systemic sclerosis, discoid lupus erythematosus, and diffuse large B-cell lymphoma in the context of pro-inflammatory chemokines.

## 2. Materials and Methods

A medical literature search of PubMed (1991–present) conducted in the winter of 2024 was performed using appropriate terms without date limitations. The main subject of the research was to identify the potential link between psoriasis and metabolic syndrome, with a focus on proinflammatory chemokines. Medical subject headline terms included “psoriasis and RANTES”, “skin disorders and RANTES”, “skin and RANTES”, “metabolic syndrome and RANTES”, “psoriasis and eotaxin”, “skin disorders and eotaxin”, “skin and eotaxin”, “metabolic syndrome and eotaxin”, “psoriasis and GRO-alpha”, “skin disorders and GRO-alpha”, “skins and GRO-alpha”, “metabolic syndrome and GRO-alpha”, “psoriasis and MCP-3”, “skin disorders and MCP-3”, “skin and MCP-3”, “metabolic syndrome and MCP-3”, “psoriasis and SDF-1α”, “skin disorders and SDF-1α”, “skin and SDF-1α”, “metabolic syndrome and SDF-1α”, “psoriasis and MCP-1”, “skin disorders and MCP-1”, “skin and MCP-1”, and “metabolic syndrome and MCP-1”. Duplicated publications and articles with low clinical significance were all excluded from the analysis. Originally, human and animal studies were included in the narrative review. The results of the search strings were merged, and duplicates were removed. Afterwards, the titles and abstracts of the remaining studies were screened to identify relevant articles that addressed the review subject. Afterwards, the titles and abstracts of the remaining studies were independently screened by two reviewers (M.M. and H.M.) to identify relevant articles that addressed the review subject. Disagreements between reviewers were resolved by the opinion of a fourth reviewer (A.C.). Finally, the selected eligible articles were fully reviewed.

## 3. Discussion

In this review, we focused our paper on chemokines (eotaxin, RANTES (CCL5), GROα, SDF-1α (CXCL12), MCP-1, and MCP-3 (CCL7)), which can be a crucial link between various skin diseases—including psoriasis, which is the most common dermatosis worldwide—and metabolic syndrome. For instance, psoriasis and obesity constitute a bidirectional, synergistic inflammatory cycle, where obesity acts as a chronic, low-grade inflammatory state that directly aggravates psoriatic skin inflammation. While adipose-derived inflammation accelerates skin inflammation, chronic inflammation from psoriasis can, in turn, facilitate insulin resistance and metabolic dysfunction, creating a “vicious cycle” of worsening both conditions. Integrated treatment must target both pathways simultaneously, with weight loss, GLP-1 agonists, and specific adipokine modulation showing the greatest potential to bridge this data gap. A comprehensive understanding of the role of selected chemokines in multiple skin disorders is essential for expanding novel modalities. This review presents insights into the molecular mechanisms underlying complex diseases and provides evidence for targeted therapies, which can effectively treat these conditions and improve patient outcomes. Examples of proinflammatory chemokines are presented in Table 1.

### 3.1. The Role of the Eotaxin Family Proteins in Skin Diseases

Dermal fibroblasts act as a significant, natural source of the eotaxin family (eotaxin-1, eotaxin-2, and eotaxin-3), which are CC chemokines responsible for recruiting eosinophils to inflammatory sites, such as in atopic dermatitis and psoriasis. Research by Dulkys et al. [11] indicated that when stimulated with Th2 cytokines (IL-4) or a combination of IL-4 and TNF-α, these fibroblasts produce chemokines in a specific order of mRNA expression intensity: eotaxin-1 > eotaxin-3 > eotaxin-2. These findings highlight that fibroblasts are not merely structural cells but active participants in the immune response in the skin. Dulkys et al. [11] demonstrated that dermal fibroblasts are a natural source of eotaxin 1, eotaxin-2, and eotaxin-3. The eotaxin family is a group of CC chemokines engaged in the recruitment of eosinophils to inflammation sites. An increased expression of eotaxins was observed in inflammatory skin diseases such as psoriasis and atopic dermatitis [11]. Furthermore, Owczarek et al. [12] confirmed that transcript levels of eotaxin-1, eotaxin-2, and eotaxin-3 are elevated in the lesional skin of atopic dermatitis (AD) patients, with eotaxin-3 directly correlating to disease severity. While eotaxin-3 levels in serum strongly reflect AD activity, eotaxin-2 does not always show this correlation. Both are key to eosinophil recruitment in late-phase allergic reactions. Similarly, Yawalkar et al. [13] have also observed that eotaxin expression is higher in atopic patients than in nonatopic individuals. Increased infiltration of eosinophils in the lesional skin of patients with AD is correlated with eotaxin expression. Both CCR3 immunoreactivity (protein) and mRNA expression levels are significantly elevated in both nonlesional and lesional AD skin fragments compared to controls. These findings indicate that enhanced local production of eotaxin leads to the recruitment of CCR3-expressing eosinophils and T lymphocytes, contributing to the initiation and maintenance of inflammation in AD [13] (Table 2).

Hossny et al. [14] found that elevated plasma eotaxin levels in patients with atopic dermatitis (AD) compared to those in acute urticaria (AU) are likely linked to chronic, persistent tissue eosinophilia [14]. The study suggests eotaxin serves as a biomarker for lesional activity and acts to recruit eosinophils, though levels did not correlate with age, eosinophil counts, or total IgE in patients (Table 2).

Park et al. [15] reported that topical tacrolimus applied twice daily for 8 weeks, in patients with AD, decreases the number of eosinophils in skin tissue and suppresses the expression of eotaxin. The EASI score at baseline (week 0) ranged from 10.4 to 65.2 (mean ± SD: 28.0 ± 16.6). After 8 weeks of treatment with tacrolimus, the EASI score ranged from 0.6 to 63.2 (mean ± SD: 12.2 ± 15.3). This reduction represents a statistically significant improvement in EASI scores, consistent with findings that tacrolimus ointment is highly effective for atopic dermatitis [15] (Table 2).

Frezzolini et al. [16] showed that serum levels of eotaxin are higher in patients with bullous pemphigoid (BP) than in healthy controls and patients with pemphigus vulgaris (PV). Interestingly, the highest levels of eotaxin are detected in BP blister fluids, compared to both corresponding BP sera and blister fluids from healthy controls. Strong immunostaining for eotaxin and CCR3 was spotted in BP skin tissue, mainly in lesional skin and, to a lesser extent, in perilesional skin [16]. This study indicates that the eotaxin/CCR3 pathway is a crucial part of the localized inflammatory reaction in BP, with blister fluids being a primary site of this accumulation (Table 2).

Gunther et al. [17] found that eotaxin-1 and eotaxin-3 are significantly upregulated in the serum and blister fluid of BP patients, correlating with disease severity [17]. While eotaxin-1 is expressed by dermal endothelial cells, both chemokines drive the increased density of activated eosinophils responsible for blister formation via the CCR3 receptor [17] (Table 2).

Bock et al. [18] noted that CD26, or dipeptidyl peptidase IV (DPP IV), is a membrane-bound protease expressed on CD8 and CD4 T cells. Its function is to cleave dipeptides from the N-terminus of proteins, leading to a decrease in their biological activity. CD26 is constitutively expressed on T cells and is enzymatically active. This enzyme is involved in the processing and inactivation of pro-inflammatory mediators associated with both psoriasis and AD, such as the chemokine RANTES (regulated upon activation, expressed and secreted by normal T cells) and eotaxin. The authors observed decreased expression of CD26 on CD8 lymphocytes in patients with psoriasis and AD. This suggests reduced truncation of inflammatory chemokines such as RANTES and eotaxin. In conclusion, reduced CD26 expression in AD and psoriatic patients causes an imbalance in favor of pro-inflammatory cytokines in both clinical conditions [18] (Table 2).

### 3.2. The Role of Eotaxin Protein in Metabolic Syndrome

Vasudevan et al. [19] found that obese mice and humans have increased levels of circulating eotaxin and eotaxin mRNA in visceral adipose tissue, with levels 4.7-fold higher in visceral adipose tissue than subcutaneous tissue. They also observed a positive correlation between adipose tissue eotaxin mRNA levels and serum eotaxin protein levels, suggesting adipose tissue, particularly the stromal/vascular fraction, is a source of eotaxin. Furthermore, diet-induced weight loss in humans leads to a reduction in plasma eotaxin levels, indicating interventions targeting obesity can influence systemic eotaxin levels [19] (Table 3).

In contrast to the above-mentioned study, Herder et al. [20] found no significant increase in eotaxin levels among individuals with impaired glucose tolerance (IGT) or type 2 diabetes mellitus (T2DM). While the cross-sectional study did not rule out transient eotaxin involvement, it identified moderate correlations between eotaxin and age, C-reactive protein (CRP), and interleukin 6 (IL-60 [20] (Table 3)).

Falcone et al. [21] identified that plasma eotaxin-3 and high-sensitivity C-reactive protein (hs-CRP) levels do not differ between hypertensive patients with or without diabetes, highlighting that they are independent cardiovascular risk markers [21].

Loughrey et al. [22] found that in patients with MetS, serum eotaxin-1 levels are elevated compared with healthy controls (*p* = 0.02). Furthermore, the study revealed a twofold increase in the mRNA expression of CCR5, a receptor for eotaxin-1, on CD14+ peripheral monocytes in the MetS group (*p* = 0.03). The strong association between eotaxin-1 and the waist-to-hip ratio, together with its weaker correlation with BMI, suggests that eotaxin-1 is specifically linked to central (visceral) obesity. Furthermore, eotaxin-1 was correlated with MCP-1, a chemokine known to contribute to macrophage infiltration and the development of insulin resistance, as reflected by HOMA-IR and fasting insulin levels. These outcomes may indicate that eotaxin-1 may play a role in the inflammatory processes underlying insulin resistance. Additionally, atorvastatin treatment reduces both circulating eotaxin-1 levels (*p* < 0.05) and CCR5 mRNA expression in these monocytes (*p* = 0.02) [22] (Table 3).

### 3.3. The Role of Growth-Regulated Peptide (GRO-α)/CXCL1 in the Skin

Growth-regulated peptide (GRO-α) is a chemotactic agent for neutrophils and promotes migration and proliferation of keratinocytes as well as angiogenesis in cutaneous wound healing. Interestingly, inactive keratinocytes produce little GRO-α. However, stimulation with tumor necrosis factor-α (TNF-α) increases GRO-α mRNA expression and protein production in oral keratinocytes and normal human skin cells (*p* < 0.001). Oral keratinocytes exhibit a higher response than normal human skin cells (*p* < 0.01). Additionally, interleukin-1α (*p* < 0.005) and interleukin-4 (*p* < 0.01) stimulation also induce production of amounts of GRO-α in oral, but not skin keratinocytes, and there is a synergistic effect on GRO-α production when oral keratinocytes are stimulated with combinations of interleukin-1 (IL-1) and TNF-α or IL-4 and TNF-α. Notably, none of these cells responds to interferon-γ. One of the functions of GRO-α in keratinocytes is to selectively recruit neutrophils in mucocutaneous inflammatory diseases [23] (Table 4).

### 3.4. The Role of GRO-α in Skin Diseases

Schaeper-Gerhardt et al. [24] found GRO-α expression is elevated in cutaneous squamous cell carcinoma (cSCC) cell lines compared to normal keratinocytes, and is also detectable in human cSCC tissue. Furthermore, reducing GRO-α expression through knockdown in a skin equivalent model decreases tumorigenicity (GRO-α lacks the ELR-motif, which determines angiogenic features and elevates the dissemination of CXCR4-positive tumor cells to distant organs), and the addition of sirolimus, an mTOR inhibitor, reduces GRO-α expression in both keratinocytes and cancer cell lines. The researchers concluded GRO-α may play a key role in human keratinocyte carcinogenesis, and it may represent a molecular mechanism for the preventive effect of mammalian target of rapamycin (mTOR) inhibitors, such as sirolimus, in cSCC development and progression [24] (Table 5).

Kojima et al. [25] showed that the *GRO-α* gene is selectively overexpressed in psoriatic lesions, strongly suggesting that the overexpression of GRO-α is a response of keratinocytes to activated T cells in psoriasis. There was no significant change in the PASI after 1 week of cyclosporin A (CsA) therapy, either in the overall cohort of treated patients or in the subgroup included in this study. It is consistent with findings that GRO-α expression is downregulated by cyclosporin A in vivo, but is not directly suppressed in cultured keratinocytes in vitro. However, the precise mechanism responsible for the selective overexpression of GRO-α in psoriatic lesions remains unknown [25] (Table 5).

Gillitzer et al. [26] noted that GRO-α and IL-8 mRNA are produced by different cell populations and are differentially expressed in psoriatic lesions. Interestingly, GRO-α and interleukin-8 (IL-8) may influence different steps of neutrophil migration (diapedesis from the blood and further movement to the higher epidermal layer) and activation in vivo. Specifically, GRO-α is uniquely found in psoriatic skin, particularly the papillary dermis, and is expressed in vessel-associated cells, while it is absent in normal and perilesional skin. GRO-α appears to enhance neutrophil activation and movement out of blood vessels and into the skin [26] (Table 5).

Konig et al. [27] found that GRO-α promotes T cell chemotaxis in vitro and can also promote migration and accumulation of T cells in vivo. GRO-α mRNA was predominantly expressed by monocytic cells (KIM6^+^). Its expression was restricted exclusively to the synovial lining layer in patients with rheumatoid arthritis (RA) and psoriatic arthritis (PsA) [27] (Table 5).

Kulke et al. [28] found that increased levels of GRO-α immunoreactivity could be detected in the suprabasal layers of psoriatic lesions. Additionally, chemokine activation of target cells is mediated by specific receptors, including two CXCR receptors, which have similar affinity for interleukin-8 but different affinities for GRO-α. Overexpression of CXCR2 mRNA in psoriatic epidermis is pronounced, although lower levels of expression are also detectable in normal epidermis. No significant differences in expression pattern or level were observed between early lesions (duration 2–3 weeks) and chronic plaque-type lesions (duration approximately 2–3 months). Moreover, CXCR2 appears to be the predominant receptor expressed in lesioned psoriatic keratinocytes, whereas the low levels of CXCR1 detected may be attributable to infiltrating neutrophils. These results suggest that increased CXCR2 activation in psoriatic epidermis may contribute to altered keratinocyte differentiation [28] (Table 5).

Raychaudhuri et al. [29] also showed that GRO-α activity is increased in the keratinocytes of psoriatic lesions [29], which contributes to the influx of neutrophils, a key feature of psoriasis. Specifically, Raychaudhuri et al. [31] used in situ hybridisation to detect increased GRO-α mRNA in the differentiated layers of psoriatic epidermis, associating it with keratinocytes. Furthermore, they found that IL-8, another neutrophil-activating peptide, was also upregulated in these areas, indicating a coordinated inflammatory response involving these chemokines. This interplay between keratinocytes and immune cells is a central aspect of psoriasis pathogenesis [29] (Table 5).

Zeng et al. [30] also found that MBL deficiency impairs GRO-α chemokine production in skin keratinocytes after IMQ activation, which may explain the reduced neutrophil recruitment in the skin. Additionally, they showed that MBL protein activates IMQ-induced GRO-α expression and the mitogen-activated protein kinase/nuclear factor kappa B (MAPK/NF-κB) signaling pathway in human keratinocyte cell lines (HaCaT cells) in vitro. Additionally, they found that MBL deficiency impairs GRO-α chemokine production in skin keratinocytes after IMQ activation, which may explain the reduced neutrophil recruitment in the skin [30] (Table 5).

Kato et al. [31] found that serum GRO-α levels are markedly elevated in psoriasis during acute exacerbation and pustule formation (447.2 pg/mL) and decreased following systemic corticosteroid therapy (153.8 pg/mL). However, serum GRO-α levels increase again at the time of pustule relapse (310.7 pg/mL). Moreover, serum GRO-α levels correlate with CRP levels, but the correlation between serum GRO-α levels and circulating neutrophil or leukocyte counts is unclear due to the patient’s treatment with systemic corticosteroids. Serum GRO-α levels in patients with generalized pustular psoriasis (GPP) are also higher than in patients with psoriasis vulgaris (PsV) or palmoplantar pustulosis (PPP) [31] (Table 5).

### 3.5. The Role of GRO-α in Metabolic Syndrome

Takashi et al. [32] showed elevated serum GROα levels in patients with type 1 diabetes mellitus (T1DM). Specifically, serum GROα levels were higher in subjects with acute-onset [median 113.2 ng/mL (41.75–457.2)] or slowly progressive [median 100.8 ng/mL (32.87–225.0)] T1DM (SPT1DM) than in patients with T2DM [median 71.58 ng/mL (32.45–152.6); *p* = 0.01 and *p* = 0.03, respectively]. In a subpopulation of patients with slowly progressive T1DM and preserved β-cell function, the decrease in fasting C-peptide concentration over the year correlated with GROα concentration in those who retained preserved β-cell function (*n* = 11; r^2^ = 0.524, *p* = 0.012). Serum GROα levels predicted the next loss of endogenous insulin secretion in patients suffering from SPT1DM, possibly reflecting ongoing anti-islet autoimmune activity [32] (Table 6).

Feng et al. [33] found that urinary sediment GRO-α mRNA levels were upregulated in patients with diabetic neuropathy and were linked to declining renal function and the degree of renal interstitial fibrosis. Furthermore, these mRNA levels were negatively correlated with estimated glomerular filtration rate (eGFR), with a correlation coefficient (r) of −0.2275 and a *p*-value of 0.0301. This suggests a potential role for GRO-α mRNA as a biomarker in monitoring diabetic nephropathy progression and its impact on kidney health [33] (Table 6).

Darakhshan et al. [34] observed that mothers with gestational diabetes mellitus (GDM) have elevated serum levels of GROα, and this increase is also seen in their newborns. It may be a relationship between TNF-α production as an inflammatory mediator and endothelial damage in GDM, highlighting a possible link between elevated levels of angiogenic chemokines such as GROα and endothelial dysfunction in GDM patients. This finding suggests a potential link between GROα and the development or progression of GDM, as well as its impact on the fetus [34] (Table 6).

Sajadi et al. [35] demonstrated that elevated serum GROα levels in T2DM act as markers of chronic inflammation and tissue stress, correlating with vascular damage repair attempts. This increased GROα, largely from hypertrophied adipocytes, drives macrophage and neutrophil recruitment to adipose tissue, amplifying inflammation and systemic insulin resistance [35] (Table 6).

Nunemaker et al. [36] reported that circulating GROα levels increase with the onset of T2DM. Additionally, GROα is produced by pancreatic islets under stress and can negatively impact islet function, potentially contributing to T2DM development. Serum levels of GRO-α were significantly higher in hyperglycemic mice (blood glucose > 400 mg/dL, *n* = 5) than in non-hyperglycemic mice (blood glucose < 250 mg/dL, *n* = 8; *p* < 0.001). In pancreatic islets, GRO-α was highly sensitive to low levels of inflammation, and GRO-α appeared capable of synergistically affecting islet function. This data suggested that GRO-α was a marker of islet dysfunction and could be a key contributor to islet failure in T2DM. Subsequently, it was spotted that free fatty acids upregulated GRO-α expression, consistent with palmitate-induced GRO-α expression observed in human islets [36] (Table 6).

In contrast, Johny et al. [37] found that patients with T2DM and coronary artery disease (CAD) had decreased GROα levels. CD4^+^ T cells, which play a key role in the immunopathogenesis of plaque in atherosclerosis, were positively correlated with eotaxin-1 levels, whereas CD8^+^ T cells were negatively correlated with GRO-α [39] (Table 5). These results contrast with the above finding, where elevated GROα levels are associated with hyperglycemia. The authors speculated this may be a compensatory response to T2DM with coronary atherosclerosis [37] (Table 6).

Tang et al. [38] observed that GROα is produced in the retinas of diabetic mice upon incubation with Toll-like receptor 2 (TLR2) agonist Pam3CysK, but incubation with other TLR ligands or IL-1β did not boost cytokine production in the retinas of diabetic or nondiabetic mice [38] (Table 6).

Wang et al. [39] found that an angiotensin II-induced influx of monocytes into heart tissue is primarily mediated by the GROα-CXCR2 (C-X-C motif chemokine receptor 2) signaling pathway, which exacerbates cardiac remodeling. The study suggests inhibiting GROα and/or CXCR2 could be a potential therapeutic strategy for hypertensive heart disease by targeting the process that initiates and worsens cardiac remodeling [39] (Table 6).

Camnitz et al. [40] demonstrated that GROα levels are highest in patients with hyperlipidemia who are not receiving statin therapy. This is likely due to a reduction in *GROα* gene expression in peripheral blood mononuclear cells (PBMCs) in patients receiving statins [40] (Table 6).

Akhtar et al. [41] observed that partial carotid artery ligation in apolipoprotein E knockout mice reduced atherosclerotic plaque formation, macrophage accumulation, and GROα expression in endothelial cells. Furthermore, the study revealed endothelial hypoxia-inducible factor-1α promoted atherosclerosis by increasing GROα expression, which is mediated by microRNA-19a, and subsequently enhanced monocyte adhesion [41]. These findings suggest a potential therapeutic target for atherosclerosis by blocking GROα production (Table 6).

### 3.6. The Role of Monocyte Chemoattractant Protein-1 (MCP-1)/CCL2 in Skin

Yamashiro et al. [42] observed that monocyte chemoattractant protein-1 (MCP-1) enhances the recruitment of circulating monocytes to the skin as well as their differentiation into macrophages. Although the effect was transient, the influx of monocytes into the skin did not result in tissue damage [42] (Table 7).

Kunstfeld et al. [43] discovered that MCP-1 recruited more human T cells into human skin grafts than recombinant RANTES, SDF-1, or IP-10. This study highlighted that MCP-1 plays a role as a T cell chemoattractant in skin inflammatory conditions [43] (Table 7).

### 3.7. The Role of Monocyte Chemoattractant Protein-1 (MCP-1) in Skin Diseases

Nakamura et al. [44] reported that MCP-1 alone is not sufficient to trigger an inflammatory response, but its presence can modulate the inflammatory and immune responses initiated by exogenous stimuli. MCP-1 is a key player in recruiting and activating these immune cells, particularly monocytes, basophils, and T cells, to sites of inflammation or tissue damage. Moreover, MCP-1 facilitates the migration and accumulation of antigen-presenting cells in the epidermis by acting as a chemotactic signal from keratinocytes. Nonetheless, MCP-1 expression alone did not trigger spontaneous skin inflammation. It did modify inflammatory responses when challenges (for example, contact hypersensitivity) were applied, indicating that MCP-1 can boost immune cell recruitment and activation in skin immune responses [44] (Table 8).

Shallo et al. [45] showed that MCP-1 levels were elevated in the burned skin of both young and old mice 1 day after burn injury compared with sham mice. However, MCP-1 levels in old burn mice were approximately 50% lower than in young burned mice. This may be due to the different wound types (burns, excisions, and human and mouse wounds). Nevertheless, MCP-1 levels in old mice reached the same level as in young mice 4 days after burn injury. Interestingly, no difference in wound macrophage accumulation was observed in young and old mice at any time point. In conclusion, the difference in dermal MCP-1 levels between young and old mice is not associated with a difference in macrophage influx into the burn wound. The decrease in MCP-1 levels in old mice may affect other phases of wound healing [45] (Table 8).

Tabatabaei-Panah et al. [46] found that only the MCP-1 rs1024611 and methylene-tetrahydrofolate reductase (MTHFR) rs1801133 single-nucleotide polymorphisms (SNPs) could be linked to alopecia areata (AA) pathogenesis. This chemotactic function of MCP-1 causes the inflammatory influx seen around hair follicles in AA. However, the specific MCP-1 promoter polymorphism studied (rs1024611) was not directly associated with AA sensitivity in this cohort. The presence of the upper-mentioned variant alleles in MCP-1 and the related *MTHFR* gene was associated with monocytes and T cells at sites of inflammation and tissue injury. Summing up, these SNPs may contribute to the pathogenesis of AA by influencing MCP-1 activity [46] (Table 8).

Mehta et al. [47] found serum MCP-1 levels are elevated in psoriasis patients compared to healthy controls. MCP-1 could be a useful biomarker for psoriasis and could potentially be used to monitor disease activity and response to treatment. The emphasized MCP-1 activity provides that it contributes to the influx of monocytes, macrophages, and natural killer cells into psoriatic skin lesions. Subsequently, it might enhance local cytokine production, maintain chronic inflammation, and link cutaneous disease to broader cardiometabolic dysregulation (for instance, atherosclerosis) [47] (Table 8).

Studies by Giustizieri et al. [48] and Zablotana et al. [49] further support the role of MCP-1 in psoriasis. MCP-1 mRNA(+) keratinocytes are present in the basal layer of skin lesions, implying that MCP-1 is produced by keratinocytes and may play a role in the recruitment of inflammatory cells to the skin. MCP-1 was strongly activated by IFN-γ in cultured keratinocytes from both psoriasis and AD patients, which highlights that MCP-1 production by keratinocytes is driven by Th1-type cytokine signals. IFN-γ is typically released by activated T cells within inflamed skin. Furthermore, the MCP-1-2518 GG genotype is associated with increased psoriasis risk, indicating a genetic component to MCP-1 levels [48,49] (Table 8).

Dai et al. [50] provided further evidence that MCP-1 is a promising biomarker and therapeutic target in psoriasis. MCP-1 levels are elevated in patients with PsV and are positively correlated with PASI score, suggesting MCP-1 could be used to monitor disease activity and treatment response. Moreover, acitretin therapy reduces MCP-1 levels in patients with PsV (before treatment 266.86 ± 32.75, and after treatment 195.67 ± 28.91). MCP-1 may be involved in the mechanism of acitretin action, and targeting MCP-1 may be a novel treatment for psoriasis [50] (Table 8).

In patients with psoriasis, MCP-1 may be used as a potential local inflammatory marker and as a new tool to assess disease severity and anti-TNF-α treatment efficacy. Anti-TNF-α therapy (adalimumab/etanercept) moderately reduced plasma MCP-1 levels and robustly decreased MCP-1 expression in psoriatic skin as s reported by Lembo et al. [51] (Table 8).

### 3.8. The Role of Monocyte Chemoattractant Protein-1 (MCP-1) in Metabolic Syndrome

Malin et al. [52] indicated that MCP-1 protein levels are higher in older bariatric patients and correlate with longer time of performed surgeries and increased metabolic risk, potentially indicating a link between MCP-1, inflammation, and frailty in this population. Circulating MCP-1 levels were significantly higher in older than in younger adults (*p* = 0.04), independent of HbA_1_c. Nevertheless, serum MCP-1 was positively associated with increased metabolic risk severity (R = 0.27, *p* = 0.01). In addition, operating time was significantly correlated with both HbA_1_c (R = 0.30, *p* = 0.01) and omental MCP-1 protein content (R = 0.31, *p* < 0.01). Nevertheless, serum MCP-1 was significantly correlated with increased metabolic risk severity (R = 0.27, *p* = 0.01;, but not with BMI (R = 0.14, *p* = 0.14). These findings suggest that elevated MCP-1 is linked to inflammation and frailty, rather than just BMI [52] (Table 9).

Li et al. [53] suggested that MCP-1 is involved in the pathogenesis of both T2DM and MetS and may be particularly relevant in patients suffering from both conditions. Specifically, elevated MCP-1 levels are found in patients with T2DM-MetS, and these levels correlate positively with various cardiovascular risk factors, like BMI, waist-hip ratio, waist circumference, blood pressure, triglycerides, and HOMA-IR, suggesting that MCP-1 reflects the severity of metabolic disturbance and central obesity MCP-1 could be a useful biomarker for assessing cardiovascular risk in this population [53] (Table 9).

Xu et al. [54] suggested the MCP-1 A-2518G polymorphism may be a risk factor for macrovascular complications in patients with T2DM. These authors observed a higher frequency of this polymorphism in patients with macrovascular and microvascular complications compared to healthy individuals, implying its possible involvement in the development of these complications in the course of T2DM. Moreover, it was shown that the lower BMI and free cholesterol, as well as higher HDL-C levels, appeared to be protective factors against the development of T2DM, whereas higher levels of TG, LDL-C, and the MCP-1 A-2518G G/G genotype frequency were independent risk factors for T2DM [54] (Table 9).

Research by Oh et al. [55] indicated that therapeutic lifestyle modification (TLM) can be effective in reducing inflammation and cardiovascular risk in patients with MetS. Specifically, TLM may slow the rate of increase in MCP-1 levels, which has been linked to inflammation and cardiovascular disease [55] (Table 8). Additionally, physical exercise can reduce levels of MCP-1 and is further correlated with a decrease in visceral fat volume as described by Troseid et al. [56] (Table 9).

### 3.9. The Role of Monocyte Chemotactic Protein (MCP-3)/Chemokine (C-C Motif) Ligand 7 (CCL7) in the Skin

Monocyte chemotactic protein (MCP-3), also called chemokine (C-C motif) ligand 7 (CCL7), is a small protein that can induce chemotaxis of monocytes.

Research by Leijs et al. [57] suggests that exposure to polychlorinated biphenyls (PCBs) may increase the risk of skin inflammation, particularly through elevated levels of the pro-inflammatory chemokine MCP-3. Specifically, the study observed a moderate positive trend between the total amount of dioxin-like PCBs (DL-PCBs) and the levels of MCP-3, and further statistical analysis demonstrated that EPGN was significantly positively correlated with MCP-3 (ρ = 0.457, *p* = 0.049) [57] (Table 10).

### 3.10. The Role of Monocyte Chemotactic Protein (MCP-3)/Chemokine (C-C Motif) Ligand 7 (CCL7) in Skin Diseases

Brunner et al. [58] noticed that MCP-3 levels are elevated in patients with atopic dermatitis and correlate with the atopic dermatitis score (SCORAD) and body surface area (BSA), but not with BMI. This suggests MCP-3 plays a role in AD severity, potentially linking AD to cardiovascular issues. MCP-3, an inflammatory mediator, was also involved in atherosclerosis development. MCP-3 is a chemokine that participates in the recruitment and activation of monocytes and macrophages, being a risk factor for atherosclerosis [58] (Table 10).

Similar findings were reported by He et al. [59], who found that MCP-3 levels are elevated in pensioners with AD (>60 years old). MCP-3 may play a role in the increased risk of cardiovascular disorders in pensioners with AD. MCP-3 leads to monocyte recruitment into the vascular wall, a key step in atherogenesis. This could explain the increased levels of cardiovascular and atherosclerosis markers, like MCP-3, which is observed in older patients suffering from AD. [59] (Table 10).

Yanaba et al. [60] found that serum MCP-3 levels are higher in patients with systemic sclerosis (SSc) than in healthy controls. Additionally, MCP-3 levels are elevated in patients with diffuse cutaneous SSc (dcSSc) relative to those with limited cutaneous SSc (lcSSc). MCP-3 enhances the migration of monocytes and T cells into sick tissues, leading to persistent inflammation. Activated monocytes differentiate into macrophages, which free profibrotic cytokines (e.g., TGF-β), empowering fibroblast activation and excessive extracellular matrix deposition. Furthermore, MCP-3 levels are elevated in patients with pulmonary fibrosis and lower vital capacity (VC), and inversely correlated with %VC and carbon monoxide transfer factor (DLCO) in SSc patients. Overall, MCP-3 may be a key player in the progression of fibrosis in SSc [60] (Table 10).

Brunner et al. [61] highlighted the crucial role of MCP-3 in the pathogenesis of psoriasis, particularly its involvement in TNF-α-dependent Th1/Th17-mediated inflammation. In a mouse model of imiquimod-induced psoriasis, MCP-3 was found to influence myeloid cell inflammation, suggesting its role in orchestrating the immune response in psoriatic skin. Moreover, MCP-3 is responsible for the upregulation and secretion of pro-psoriatic cytokines, including IL-12p40, IL-17C, and CCL20. These cytokines contribute to the inflammatory cascade in psoriasis, promoting keratinocyte proliferation and immune cell recruitment. Interestingly, inactivation of MCP-3 led to an increase in the concentration of the antipsoriatic cytokine IL-4. MCP-3 may not only promote inflammation but also suppress anti-inflammatory mechanisms in psoriasis. In addition, in humans receiving infliximab, a TNF-α blocker, MCP-3 levels in lesional psoriatic skin are reduced within 16 h of a single intravenous infusion. This further supports the link between MCP-3 and TNF-α-mediated inflammation in psoriasis [61] (Table 10).

Similarly, Barbarroja et al. [62] showed that MCP-3 levels are elevated in the synovial fluid of patients with active PsA compared to those with osteoarthritis. MCP-3 may play a role in the inflammatory processes and joint damage associated with PsA. MCP-3 attracts monocytes, macrophages, T lymphocytes, and dendritic cells into the synovial part of the joint via CCR1, CCR2, and CCR3 receptors. Persisting synovitis, the influx of the above-mentioned immune cells contributes to chronic synovial inflammation, a hallmark of PsA [62] (Table 10).

### 3.11. The Role of MCP-3 in the Metabolic Syndrome

A study by Bradley et al. [63] suggests that MCP-3 may play a role in the development of autoimmune diabetes by attracting Th1 cells to the pancreas, where they can damage insulin-producing beta cells. MCP-3 is manufactured in a Th1-dominant immune response, which is strongly linked to the pathogenesis of T1D. MCP-3 acts as a chemoattractant for monocytes, macrophages, and T lymphocytes, promoting their migration into pancreatic islets and amplifying local inflammation. The authors suggest that blocking MCP-3 (or its associated receptors) may prevent the infiltration of Th1 cells, potentially protecting cells from autoimmune attack and halting the development of diabetes [63].

Additionally, Jiao et al. [64] showed that MCP-3 may be implicated in the development of obesity. Elevated FFAs in obesity strongly activate MCP-3 expression in adipocytes, which connects metabolic excess to inflammatory signaling. MCP-3 upregulation is mediated through activation of NF-κB (via IKKβ) and c-Jun NH_2_-terminal kinase (JNK) pathways, which are modulators of inflammation and insulin resistance. In addition to infiltration and inflammatory cytokine production, MCP-3 participates in chronic adipose tissue inflammation, which impairs insulin signaling and contributes to metabolic dysfunction [64] (Table 11).

Interestingly, Lan et al. [65] found that MCP-3 levels may be a predictor of pregnancy-induced hypertension (PIH). Reduced MCP-3 may lead to defective trophoblast invasion and incorrect placental development, leading to endothelial dysfunction and hypertension later in pregnancy. Moreover, the authors showed that MCP-3 levels in the first trimester were negatively associated with the further development of PIH. This study implies that monitoring MCP-3 could aid in identifying women at risk for PIH, a condition that can lead to serious complications like preeclampsia and eclampsia [65] (Table 11).

### 3.12. The Role of SDF-1α (CXCL12) in the Skin

The study by Zheng et al. [66] showed that C-X-C motif chemokine 12 (CXCL12) is expressed in dermal fibroblasts (DFs) and its expression increases during the telogen and catagen stages of the hair cycle, which suggests CXCL12’s involvement in hair follicle regression. The inhibitory effect of CXCL12 is mediated mainly through CXCR4 activation, rather than CXCR7. Blocking CXCR4 significantly promotes hair growth. CXCL12–CXCR4 interaction activates intracellular STAT3 and STAT5 signaling pathways, which suppress the proliferation of hair follicle cells and inhibit hair growth [66] (Table 12).

### 3.13. The Role of SDF-1α (CXCL12) in Skin Diseases

Inhibiting the CXCL12/CXCR4 signaling pathway holds promise for treating hair loss. Specifically, a study by Zheng et al. [66] indicated that CXCL12 inhibits hair growth by activating the CXCR4 receptor and the STAT pathway; thus, blocking this pathway could promote hair growth. Dermal fibroblasts (DFs) produce CXCL12, which then interacts with CXCR4 on the outer root sheath cells (ORS) and dermal papilla (DP) of hair follicles. This interaction, when activated, slows hair growth and induces follicle regression. Blocking this pathway, potentially using antibodies or small-molecule inhibitors, could counteract these effects [66] (Table 12).

Guo et al. [67] provided strong evidence that the CXCL12/CXCR4 signaling pathway plays a key role in wound healing. CXCL12 levels are elevated in the peripheral areas of wounds, and CXCR4 is expressed on both epithelial cells and epidermal stem cells. Blocking the CXCL12/CXCR4 axis reduces epidermal stem cell migration and wound healing in vivo, while CXCL12 treatment promotes epidermal stem cell proliferation and migration and accelerates wound healing. These findings suggest CXCL12/CXCR4 signaling is essential for wound healing by promoting the migration and proliferation of epidermal stem cells. This makes CXCL12/CXCR4 signaling a promising target for the development of new regenerative therapies for wound healing [67] (Table 12).

Cao et al. [68] provided evidence that the CXCL12/CXCR4 signaling pathway is involved in radiation-induced skin injury and fibrosis. CXCL12 and CXCR4 expression are increased in irradiated skin, and blocking this pathway with AMD3100, a CXCL12/CXCR4 inhibitor, reduces radiation-induced skin damage and fibrosis in rats. CXCL12/CXCR4 pathway promotes the migration and proliferation of inflammatory cells and fibroblasts, contributing to the development of radiation-induced skin issues [68] (Table 12).

Zhang et al. [69] and Luo et al. [70] provided evidence that CXCL12 can be used to promote wound healing and angiogenesis. Zhang et al. [69] showed that CXCL12 can promote chemotaxis of endothelial progenitor cells (EPCs) to the wound site, which can increase angiogenesis in the graft area. CXCL12 can be used as a new therapeutic option to promote tissue vascularisation in transplanted organs and chronic ischemic wounds [69] (Table 12). Likewise, Luo et al. [70] showed that a modified mRNA (modRNA) CXCL12 can promote wound healing in small skin lesions and reduce scar formation and angiogenesis in the subcutaneous layer of the skin. ModRNA-CXCL12 could be used to develop new treatments for chronic wounds and other conditions requiring wound healing and tissue regeneration. The CXCL12/CXCR4 axis takes part in intracellular pathways that enhance epithelialization, decrease apoptosis, and coordinate inflammatory responses, which are obligatory in efficient wound healing and scar formation [70] (Table 12).

A study by Sun et al. [71] highlighted the role of the CXCR4/CXCL12 signaling pathway in the development of AD. Skin-resident NKT cells, which express CXCR4, are drawn to sites of inflammation by CXCL12 produced by fibroblasts. This interaction leads to the formation of NKT cells and fibroblast clusters during allergic reactions like AD. In mouse models dealing with AD, NKT cells penetrated into the skin and made inflammatory cytokines, like IL-4, IFN-γ, and IL-17, boosting both acute and chronic skin inflammation. Elevated levels of CXCR4 + Vα24Jα18 + NKT cells and CXCL12 in AD skin suggest the CXCR4/CXCL12 pathway is crucial for recruiting these cells to inflamed skin tissue [71] (Table 12).

Bernat-Peguera et al. [72] reported that platelet-derived growth factor receptor (PDGFR)/CXCL12 interaction, activated at the late stages of human and mouse skin SCC, promotes autocrine CXCL12/CXCR4 signaling, tumor cell invasion, and tumor metastasis. The researchers found that PDGFR and CXCL12 are co-expressed in SCC cells and PDGFR activation stimulates CXCL12 production. Moreover, CXCL12/CXCR4 signaling promotes tumor cell migration and invasion in SCC cells. In addition, blocking either the PDGFR or CXCL12/CXCR4 pathway inhibits tumor cell migration and invasion in SCC cells [72] (Table 12).

### 3.14. The Role of SDF-1α (CXCL12) in the Psoriasis

Skrzeczynska-Moncznik et al. [73] found CXCL12 and chemerin work together to influence the movement of plasmacytoid dendritic cells (pDCs) into psoriatic skin. pDCs, crucial for innate immunity and type I interferon production, express both CXCR4 (which binds CXCL12) and CMKLR1 (which binds chemerin). Both CXCL12 and chemerin are present in psoriatic skin, and blocking either chemokine’s signaling reduces pDC influx, leading to alleviating psoriatic skin lesions [73] (Table 12).

Abdelaal et al. [74] noticed CXCL12 is a potential marker of disease severity in psoriatic patients. These researchers found CXCL12 expression is higher in PsA patients than in PsV patients before treatment with methotrexate (MTX). They also observed that MTX treatment decreases CXCL12 expression in PsV patients. This depletion was paralleled by clinical improvement, as shown by decreases in PASI score, and corresponded moderately with the percentage decrease in CXCL12 expression. CXCL12 may play a role in the development and progression of PsV and PsA, and, therefore, CXCL12 may be a marker of disease severity in psoriatic patients [74] (Table 12).

### 3.15. The Role of SDF-1α (CXCL12) in the Metabolic Syndrome

A study by Jung et al. [75] found CXCL12 levels are correlated with waist circumference, BMI, and IR in adolescents. In adolescents, a lower circulating level of CXCL12 is linked to elevated waist circumference and higher BMI, suggesting that early central obesity may be connected to altered chemokine regulation. In addition, CXCL12 acts on vascular homeostasis and endothelial progenitor cell enrolment; its association with adiposity measures implicates it in early atherosclerotic and cardiometabolic processes even before overt disease onset. CXCL12 could be a biomarker to identify adolescents at risk of developing obesity-related diseases [75] (Table 13).

Similarly, Li et al. [76] found CXCL12 levels are higher in patients with borderline high lipid profile (BHLP) than in healthy controls. The positive correlation between SDF-1α and HDL-cholesterol noted in female patients points out that CXCL12’s link to lipid fractions may be different regarding sex, emphasizing the complex regulatory mechanisms that modulate its expression in metabolic conditions [76] (Table 13).

In mice on a high-fat diet, the level of CXCL12 in white adipose tissue (WAT) is significantly higher in comparison to lean controls. Elevated CXCL12 leads to the influx of macrophages in this tissue, where they become a major source of pro-inflammatory cytokines and establish a local inflammatory state in adipose tissue. In obese mice, blocking the CXCL12 receptor (CXCR4) with an antagonist led to reduced macrophage accumulation in adipose tissue, lowered pro-inflammatory cytokine production locally, and improved systemic insulin sensitivity. The CXCL12/CXCR4 pathway plays a role in the development or progression of insulin resistance and T2DM, which are strongly linked to obesity [77] (Table 13).

A study by Liu et al. [78] found specific alleles of the *CXCL12* gene are linked to a reduced risk of hypertension in the Chinese Han population. CXCL12 plays a key role in inflammatory signaling, vascular cell migration, and immune system responses, which are a background of blood pressure regulation and vascular remodeling. Interestingly, several variants in CXCL12—specifically rs1065297, rs4948878, and rs10793538—were significantly associated with reduced risk of hypertension. CXCL12 could potentially play a role in preventing hypertension [78] (Table 13).

Research by Modanwal et al. [79] indicates the *CXCL12* gene product is associated with increased stress in obese individuals and may play a role in obesity’s development and progression. CXCL12 was described as a target for molecular docking and virtual screening along with coumarin derivatives to discover potential inhibitors that could perform as therapeutic agents in both obesity and cancer. Additionally, the same gene is linked to cancer progression, suggesting a potential therapeutic target for cancer treatment [79] (Table 13).

Aboumrad et al. [80] found that the CXCL12/CXCR4 pathway may have a protective effect on the development of autoimmune diabetes. Blocking the CXCL12/CXCR4 interaction might prevent immune cells from reaching the beta cells of the pancreas, and subsequently the development of type 1 diabetes. CXCL12-attracted CXCR4^+^ T cells, transferred diabetogenic T cells into recipient mice, and the development of diabetes was completely stopped. The protective effect appears during the selection or recruitment of Th2-like CXCR4^+^ T cells that counteract pathogenic Th1 immune actions and further delay autoimmune destruction of insulin-producing β-cells. However, the reduced number of CXCR4^+^ and CXCL12-positive cells in pancreatic islets allowed more autoreactive T cells to infiltrate and damage β-cells [80] (Table 13).

### 3.16. The Role of RANTES (CCL5) in the Skin

Chemokine (C-C motif) ligand 5 (CCL5), (RANTES) (regulated on activation, normal T cell expressed and secreted), is a proinflammatory chemokine that attracts various immune cells to sites of inflammation, including T cells, eosinophils, basophils, monocytes, natural killer cells, dendritic cells, and mast cells. This recruitment is a key part of the immune response to infection and injury.

Wakugawa et al. [81] found RANTES plays a role in skin inflammation, and its production by keratinocytes (KCs) is mediated by a variety of cytokines, including TNF-α, IFN-γ, IL-1β, IL-4, and IL-13. Dexamethasone inhibits RANTES production, while tacrolimus has a partial inhibitory effect [81] (Table 14).

Matsui et al. [82] revealed RANTES plays a role in eosinophil infiltration in the skin in response to peptidoglycan (PGN), a component of the bacterial cell wall. The authors found PGN stimulates the production of RANTES by epidermal Langerhans cells, a type of dendritic cell found in the skin. RANTES then attracts eosinophils to the site of inflammation. An antibody against RANTES blocked PGN-induced eosinophil infiltration into the skin of mice. This suggests that RANTES is essential for PGN-induced eosinophil infiltration [82] (Table 14).

### 3.17. The Role of RANTES (CCL5) in Skin Diseases

Distler et al. [83] showed evidence that RANTES plays a role in the pathogenesis of SSc and fibrosis. RANTES is present in both early- and long-lasting epidermal cells of human sclerodermatous skin. RANTES can impact fibroblast activation and extracellular matrix deposition, which are the background of pathogenesis in SSc. The researchers proposed that RANTES might modulate collagen production or fibroblast function, further contributing to fibrosis development [83] (Table 14).

Bornscheuer et al. [84] indicated that RANTES does not play a role in the eosinophilic influx observed in autoimmune bullous skin diseases such as BP. They noted the absence of RANTES in blister fluids from BP patients, and that non-peptide RANTES failed to induce eosinophil influx in a BP animal model. However, in BP and related diseases where granulocytes accumulate, the authors proposed that their recruitment is less likely to be conducted by RANTES at the site of the skin lesion [84] (Table 14).

Studies by Yamada et al. [85] and Kato et al. [88] both suggested RANTES plays a role in eosinophil influx in AD. Yamada et al. [87] showed RANTES is present in both dermal and colonic tissue in patients with AD, and it is associated with the infiltration of eosinophils and T cells. RANTES attracts eosinophils and T lymphocytes—key inflammatory cells involved in AD skin lesions. The presence of RANTES mRNA, both in the skin and colon, highlights that it may lead to the recruitment of these immune cells into inflamed tissues in AD [85] (Table 14).

Likewise, Kato et al. [86] found that the number of RANTES-positive cells in the lesioned skin of AD patients is correlated with the severity of the disease. The number of RANTES^+^ cells, CCR3^+^ cells, CCR5^+^ cells, EG2^+^ cells, and CD3^+^ cells was all significantly increased in challenged lesioned skin of AD patients compared with unaffected lesioned skin and normal skin. The amount of these cells in non-affected AD skin was also higher than that in normal skin. The number of EG2^+^ cells in non-affected AD lesional skin correlated with both the peripheral blood eosinophil count and the SCORAD index. In affected lesioned skin, the amount of EG2^+^ cells correlated with the number of CCR5^+^ cells. Activated eosinophils and T cells expressed RANTES, and varying proportions of these cells were CCR3^+^ and CCR5^+^ in both affected and non-affected lesioned skin [86] (Table 14).

Gambichler et al. [87] showed CCL5/RANTES is elevated in morphea, a type of localized scleroderma. Raised expression of RANTES in morphea lesions likely shows boosted recruitment and accumulation of immune cells in the skin, a feature of the localized scleroderma inflammatory response [87] (Table 14).

Puxeddu et al. [88] reported that CCL5/RANTES is elevated in chronic spontaneous urticaria (CSU) and may play a role in its pathogenesis. CCL5/RANTES is a potent chemoattractant for T cells, eosinophils, and monocytes and plays a crucial role in the release of histamine and serotonin by mast cells. Levels of CCL5/RANTES are higher in CSU patients compared to controls (710.9 ± 25.58 vs. 512.7 ± 36.93 ng/mL; *p* < 0.0001). Nevertheless, the authors also concluded that CCL5/RANTES levels do not reflect CSU disease activity and cannot predict response to H1 antihistamine therapy [88] (Table 14).

Chong et al. [89] reported that RANTES is overexpressed in skin patients with discoid lupus erythematosus (DLE). Moreover, the authors found RANTES mRNA levels are increased in DLE skin compared with normal skin, and CD163+ macrophages, a type of macrophage that produces RANTES, are increased in perivascular areas and the epidermal–dermal junction of DLE skin [89] (Table 14).

### 3.18. The Role of RANTES (CCL5) in Psoriasis

Raychaudhuri et al. [29] point out that RANTES is crucial in the inflammatory process of psoriasis. Psoriatic KCs produce elevated levels of RANTES compared to healthy KCs, and this increased RANTES production is associated with an intensified influx of T cells to the psoriatic epidermis. Taken together, these data indicate RANTES might play a key role in the inflammatory process of psoriasis [29] (Table 14).

Fukuoka et al. [90] found that RANTES is involved in the inflammatory process of psoriasis, and active vitamin D3 may reduce RANTES production in keratinocytes. RANTES is produced by keratinocytes due to the influence of TNF-α and IFN-γ. Additionally, it acts as a chemoattractant for T lymphocytes and other immune cells, promoting their migration into the skin and stabilizing chronic inflammation in psoriasis. Specifically, they observed that RANTES is expressed in psoriatic plaques, but not in healthy skin, suggesting its role in the disease. Furthermore, they showed that tacalcitol, an active vitamin D3 analog, inhibits RANTES production in keratinocytes [90] (Table 14).

Johansen et al. [91] noted RANTES expression is elevated in lesional compared to nonlesional psoriatic skin, and there is a positive correlation between the expression of RANTES, IFNγ, and IL-17A in these lesions. The authors also found a signal transducer and activator of transcription (STAT2), which is involved in psoriasis pathogenesis by regulating the expression of RANTES and attracting IFNγ-producing immune cells to the skin. RANTES expression was significantly higher in lesioned psoriatic skin (circa four-fold increase) in comparison to non-lesioned psoriatic skin. Moreover, in the study, the authors observed a positive correlation between RANTES and IFN-γ expression in psoriatic skin lesions. These findings suggest RANTES plays a key role in the inflammatory process in psoriasis [91] (Table 14).

Kono et al. [92] described CCR5+ invariant natural killer T (iNKT) cells, which express the receptor for RANTES and may be a useful target for the development of new treatments for PsV. The authors presented that CCL5 is strongly expressed in endothelial cells of capillary veins in psoriatic dermal papillae. The number of CCR5^+^ iNKT cells is correlated with the number of endothelial cells localized in capillary veins in psoriatic skin. They also found CCR5^+^ iNKT cells expressed RANTES. In addition, the number of RANTES+ capillary veins is correlated with the maximum rete ridge length (epidermal hyperplasia), microabscess, and disease severity [92] (Table 14).

Rateb et al. [93] showed pre-treatment RANTES mRNA expression levels may be a useful marker for narrow-band ultraviolet B (NB-UVB) phototherapy efficacy and clinical improvement in patients with psoriasis. NB-UVB phototherapy reduces RANTES mRNA expression in psoriatic lesions. Moreover, there was a negative correlation between pre-treatment RANTES mRNA expression and PASI score after phototherapy, indicating that patients with higher pre-treatment RANTES mRNA expression had a greater improvement in their psoriasis symptoms after phototherapy. In addition, the authors found a negative correlation between pre-treatment RANTES mRNA expression and both the number of initial response sessions and the total NB-UVB dose at the end of phototherapy. Patients with higher pre-treatment RANTES mRNA expression require more treatment sessions and a higher total NB-UVB dose to achieve a clinical response [93] (Table 14).

A study by Joshi et al. [94] showed a decrease in serum RANTES concentration in patients with PsV compared to healthy controls. No significant association between RANTES levels and PASI score was observed. Moreover, RANTES is not a reliable biomarker of disease severity in serum. The researcher presumed that the reason for the lower level of RANTES in the psoriatic group might be redistribution from the circulation to psoriatic skin lesions, indicating its role in local inflammation rather than serving as a reliable systemic biomarker of disease activity [94] (Table 14).

This result is inconsistent with the results of the studies mentioned above, which showed RANTES expression is increased in psoriatic skin.

### 3.19. The Role of RANTES (CCL5) in the Metabolic Syndrome

Ueba et al. [95] revealed RANTES may function as a marker of atherosclerosis in healthy younger men. Plasma RANTES levels were significantly associated with MetS (the adjusted OR for age (40) was 3.4 (1.04–4.81, P 0.04)). Plasma RANTES levels are correlated with fasting glucose, diastolic blood pressure (DBP), IL-6, and platelet-derived microparticles (PDMPs), all of which are involved in the development and progression of atherosclerosis. Raised RANTES levels can lead to early atherosclerosis by enhancing interactions between activated platelets, leukocytes, and the vascular endothelium [95] (Table 15).

Gurkan et al. [96] reported RANTES may play a role in the association between MetS and gingivitis. MetS patients with gingivitis show elevated RANTES levels in their gingival crevicular fluid (GCF), despite having lower systemic inflammation. The authors hypothesize that the lower levels of systemic inflammation in MetS patients with gingivitis may lead to changes in GCF RANTES levels. This is because systemic inflammation can suppress the production of RANTES. When systemic inflammation is low, RANTES production may increase, leading to elevated levels of RANTES in the GCF. The elevated levels of RANTES in the GCF of MetS patients with gingivitis may contribute to the increased gingival inflammation seen in these patients. RANTES attracts leukocytes, T cells, and macrophages to the gingival tissue, which boosts the inflammatory infiltrate in gingivitis. RANTES may be a useful biomarker for monitoring the progression of gingivitis in MetS patients [96] (Table 15).

Herder et al. [97] found a link between higher systemic levels of RANTES and an increased risk of developing T2DM. Specifically, individuals with the highest RANTES levels have a 2.6 times higher risk of developing T2DM compared to those with lower levels. RANTES may play a role in T2DM progression, potentially through its association with pro-diabetogenic inflammatory features. Further research is needed to clarify the mechanisms involved [97] (Table 15).

Herrera-May et al. [98] found a link between the CCL5-403 G/A and CCL5-109 G/A polymorphisms and the risk of developing acute coronary syndrome (ACS). RANTES drives immune cell infiltration (monocytes, lymphocytes, etc.) into arterial walls, promoting vascular inflammation. Subsequently, enhancing production of plaque formation and progression of ACS. They also found that these polymorphisms are associated with a reduced quantity of RANTES in the Mexican population [98] (Table 15).

Dworacka et al. [99] presented the link between RANTES and glycemic control in T2DM patients. Patients with T2DM have higher serum levels of RANTES, greater postprandial and fasting hyperglycemia, as well as higher levels of 1,5 anhydroglucitol (1,5 AG) and glycated hemoglobin (HbA1c). It may be involved in the development of IR and hyperglycemia in T2DM patients [99] (Table 15).

Tokarz et al. [100] emphasized the role of RANTES and CCR5-positive microvesicles (MVs) in the development and progression of diabetic retinopathy (DR). Patients with DR show higher levels of RANTES and CCR5-positive MVs in their blood than patients without DR. Moreover, the levels of RANTES and CCR5-positive MVs correlate with the severity of DR. RANTES and CCR5-positive MVs may be involved in the inflammatory and angiogenic processes underlying DR [100] (Table 15).

Zhang et al. [101] indicated that elevated levels of RANTES can promote diffuse large B-cell lymphoma (DLBCL) in mice with T2DM. DLBCL cells cultured in high glucose conditions (30 mmol/L) showed greater RANTES expression than in a normal glucose (5 mmol/L) environment, which indicates that hyperglycaemia enhances RANTES production in lymphoma cells. Lymphoma cells that overexpress RANTES led to tumors more frequently and rapidly than cells with normal or low RANTES [101] (Table 15).

## 4. Conclusions

The association between chemokines and metabolic syndrome has emerged as a critical focus in the study of chronic inflammatory skin diseases, particularly psoriasis and atopic dermatitis. Available data indicate that these small signaling proteins do not just orchestrate local skin inflammation but also facilitate systemic inflammation, linking cutaneous flares to metabolic dysregulation such as obesity, insulin resistance, and hypertension. For instance, patients with metabolic syndrome frequently exhibit higher baseline levels of eotaxin. When these individuals also suffer from skin conditions like psoriasis or atopic dermatitis, the eotaxin pathway can become hyperactivated, exacerbating skin-fold inflammation and systemic vascular stress. Similarly, in psoriasis or hidradenitis suppurativa, elevated CXCL1 levels exacerbate the inflammatory cycle by recruiting immune cells to the skin, thereby intensifying local lesions while simultaneously contributing to the low-grade systemic inflammation that drives insulin resistance and cardiovascular risk. This connection highlights a shared pathophysiological mechanism where metabolic dysfunction amplifies the body’s inflammatory responses, potentially making skin lesions more severe and harder to treat in metabolically compromised patients.

## Figures and Tables

**Table 1:** Examples of proinflammatory chemokines are included in the manuscript.

CC Chemokines	Chemokines
MCP-1	SDF-1α (CXCL12)
MCP-3	GRO-α
RANTES (CCL5)	
Eotaxin	

**Table 2:** Summary of studies on the role of eotaxin in the skin and skin diseases, particularly psoriasis.

Author	Year	Population	Key Observation
**The Role of Eotaxin in the Skin.**			
Dulkys et al. [11]	2001	Clinical normal human skin was obtained from plastic breast surgery.	Dermal fibroblasts are a possible natural source of eotaxin.
Owczarek et al. [12]	2010	19 patients with AD	The eotaxin-1, eotaxin-2, and eotaxin-3 levels were raised in skin changes in AD patients than in non-affected skin
Yawalkar et al. [13]	1999	26 patients with AD, 11 non-atopic patients	Eotaxin increased in atopic patients in comparison to nonatopic patients.
Hossny et al. [14]	2001	16 kids with AD, 19 kids with AU, 43 healthy kids	Plasma eotaxin is higher in AD than in AU.
Park et al. [15]	2004	15 patients with AD, 15 healthy kids	Topical tacrolimus suppresses the expression of eotaxin
Frezzolini et al. [16]	2002	10 patients with active BP 3 patients with PV 10 healthy patients	The eotaxin serum levels are much higher in patients dealing with BP in comparison to healthy donors and people suffering from PV.
Gunther et al. [17]	2011	38 patients with BP 14 patients with PV 43 patients healthy	The amount of eotaxin-3 is increased in the serum and blister fluid of BP patients.
Bock et al. [18]	2001	30 patients with psoriasis 15 patients with AD 17 healthy patients	Lowered expression of CD26 on CD8 lymphocytes in patients with psoriasis and AD was due to a lowered truncation of eotaxin.

Abbreviation: AD—atopic dermatitis, AU—acute urticaria, BP—bullous pemphigoid, PV—pemphigus vulgaris.

**Table 3:** Summary of the studies on the role of eotaxin in the metabolic syndrome.

Author	Year	Population	Key Observation
**The Role of Eotaxin in the Metabolic Syndrome.**			
Vasudevan et al. [19]	2006	12 lean mice 12 obese mice 13 lean people 40 obese people	Eotaxin mRNA levels and eotaxin in visceral adipose tissue were increased in obesity in mice and humans.
Herder et al. [20]	2005	236 people with T2DM, 242 people with IGT, 244 healthy people	Correlation of eotaxin with age and key metabolic markers, for instance, with CRP, IL-6 appear to be moderate.
Falcone et al. [21]	2013	399 patients with HTN, DM, MetS	Eotaxin-3 was not different between diabetic and nondiabetic subjects. Eotaxin-3 was not associated with hs-CRP.
Loughrey et al. [22]	2012	50 patients with MetS 26 healthy people	Eotaxin-1 (*p* = 0.02) is elevated in MetS patients in comparison with healthy people.

Abbreviation: IGT—impaired glucose tolerance, T2DM—type 2 diabetes mellitus, IL-6—interleukin 6, CRP-C-reactive protein, hs-CRP—high-sensitivity C-reactive protein, HTN—hypertension, DM—diabetes mellitus.

**Table 4:** Summary of the studies on the role of GROα in the skin.

Author	Year	Population	Key Observation
**The Role of GROα in the Skin and Skin Diseases.**			
Li et al. [23]	2000	Normal human skin Oral keratinocytes	GROα may boost migration and keratinocyte proliferation.

Abbreviation: GROα—growth-regulated peptide.

**Table 5:** Summary of the studies on the role of GROα in skin diseases, particularly psoriasis.

Author	Year	Population	Key Observation
**The Role of GRO**α **in Psoriasis.**			
Schaper-Gerhardt et al. [24]	2007	Neonatal normal human epidermal keratinocytes	GROα expression is higher in cSCC cell lines in comparison to normal human keratinocytes.
Kojima et al. [25]	1993	Primary cultures of normal adult human keratinocytes; 85 chronic psoriatic patients	The *GROα* gene is selectively overexpressed in psoriatic lesions
Gillitzer et al. [26]	1996	21 psoriatic patients. 5 healthy people	GROα mRNA is strongly expressed in vessel-associated psoriatic cells of the papillary dermis.
Konig et al. [27]	2000	7 patients with rheumatoid arthritis, 8 patients with psoriatic arthritis, 10 patients with osteoarthritis.	GROα promotes T cell chemotaxis in psoriatic patients.
Kulke et al. [28]	1997	14 psoriatic patients 4 healthy people	GROα immunoreactivity could be detected in suprabasal areas of psoriatic lesions.
Raychaudhuri et al. [29]	1999	8 patients with chronic psoriatic plaque lesions, 5 patients with lichen planus, 5 patients with eczematous dermatitis, 5 healthy patients.	An increased activity of GROα is identified in the keratinocytes of psoriatic lesions.
Zeng et al. [30]	2019	MBL−/− mice	MBL deficiency restricted the chemokine GROα production from skin keratinocytes upon IMQ activation, which can be in charge of the impaired skin recruitment of neutrophils.
Kato et al. [31]	2009	15 patients with PsV, 10 patients with PPP, 19 healthy patients	Serum amount of GROα level was extremely elevated at the time of acute deterioration, with some pustular formation

Abbreviation: cSCC—cutaneous squamous cell carcinoma, GROα—growth-regulated peptide, IMQ—imiquimod.

**Table 6:** Summary of the studies on GROα in metabolic syndrome.

Author	Year	Population	Key Observation
**The Role of GRO**α **in Metabolic Syndrome.**			
Takashi et al. [32]	2011	26 subjects with acute-onset T1DM, 20 with slowly progressive T1DM, 20 with type 2 diabetes mellitus as disease controls	Serum GROα levels were higher in subjects with acute-onset or slowly progressive T1DM than in patients with T2DM.
Feng et al. [33]	2021	19 patients with DN, 25 healthy donors;	Urinary sediment of GROα mRNA is upregulated in DN patients.
Darakshan et al. [34]	2019	63 pregnant GDMM women 63 normal pregnant mothers	The serum level of GROα is increased in GDMM.
Sajadi et al. [35]	2013	100 type 2 diabetic patients; 150 healthy controls	Elevated serum levels of GROα can be found in type 2 diabetic patients with T2DM.
Nunemaker et al. [36]	2014	male BKS.Cg-*Dock7^m^* +/+ *Lepr^db^*/J (db/db) mice	GROα is increased in circulation with the onset of T2DM.
Johny et al. [37]	2021	20 healthy controls; 44 patients with T2DM; 20 patients with CAD; 38 patients with T2DM_CAD;	GROα in patients with T2DM was decreased.
Tang et al. [38]	2013	C57BL/6J mice	GROα is produced in the retinas of diabetic mice during incubation with TLR2
Wang et al. [39]	2018	WT mice (C57BL/6J, male); CXCR2 knockout mice	Angiotensin II-induced influx of monocytes in the heart tissue is mainly mediated by GROα-CXCR2 signaling, which enables and worsens cardiac remodeling.
Camnitz et al. [40]	2012	120 patients with high-dose statin; 48 patients with low-dose statin; 11 patients with no statins.	Median levels of GROα are highest in patients with hyperlipidemia who are not treated with statin therapy.
Akhtar et al. [41]	2015	Apolipoprotein E knockout mice (EC-Hif1a−/−); Control mice;	The creation process of atherosclerotic plaques, the lesional macrophage accumulation, and the expression of GROα in ECs are reduced after partial carotid ligation in ECs from apolipoprotein E knockout mice (EC-Hif1a(−/−)) in comparison to control mice.

Abbreviation: DN—diabetic neuropathy, T1DM—type 1 diabetes mellitus, GDMM—gestational diabetes mellitus mothers, T2DM—type 2 diabetes mellitus, CAD—coronary artery disease, TLR2—Toll-like receptors 2, WT—wild type, CXCR2—C-X-C motif chemokine receptor 2.

**Table 7:** Summary of the studies on the role of MCP-1 in skin.

Author	Year	Population	Key Observation
**The Role of MCP-1 in Skin and Skin Diseases.**			
Yamashiro et al. [42]	1998	Male Wistar rats	MCP-1 enhances the flow of blood monocytes and the transformation of monocytes into macrophages
Kunstfeld et al. [43]	1998	SCID mice, human skin grafts	MCP-1 boosted great quantities of human T cells into human skin grafts

Abbreviation: MCP-1—monocyte chemoattractant protein-1.

**Table 8:** Summary of the studies on the role of MCP-1 in skin diseases, particularly psoriasis.

Author	Year	Population	Key Observation
**The Role of MCP-1 in Psoriasis.**			
Nakamura et al. [44]	1995	Transgenic mice	MCP-1 alone is not able to boost an inflammatory response
Shallo et al. [45]	2003	BALB/c mice	An elevation of MCP-1 level in the burned-normal skin at 1-day post-burn in both young and aged mice, in comparison to sham-injured mice
Tabatabaei-Panah et al. [46]	2022	60 patients with alopecia areata	MCP-1 rs1024611 is associated with alopecia areata risk.
Mehta et al. [47]	2013	89 patients with psoriasis; 25 healthy people.	MCP-1 is higher in psoriatic patients in comparison to healthy people.
Giustizieri et al. [48]	2001	4 patients with psoriasis, 4 patients with atopic dermatitis, 4 healthy people.	MCP-1 mRNA(+) keratinocytes were observed in the basal layer of skin lesions in patients dealing with psoriasis.
Zablotana et al. [49]	2016	160 patients with PsV, 160 healthy people.	THE MCP-1-2518 GG genotype is linked with an elevated risk of psoriasis.
Dai et al. [50]	2014	50 patients with PsV, 50 healthy people.	MCP-1 in patients suffering from PsV was greater than in the control group.
Lembo et al. [51]	2014	30 patients with PsV, 10 healthy people.	MCP-1 might be used as a potential local inflammatory marker in patients with psoriasis.

Abbreviation: PsV—psoriasis vulgaris.

**Table 9:** Summary of the studies on the role of MCP-1 in the metabolic syndrome.

Author	Year	Population	Key Observation
**The Role of MCP-1 in the Metabolic Syndrome.**			
Malin et al. [52]	2017	55 younger ( Age: 34.9 ± 4.0 years, [BMI]: 48.2 ± 1.0 kg m^−2^); 48 older (F: 34, Age: 57.0 ± 5.1 years, BMI: 46.8 ± 1.0 kg m^−2^) adults	MCP-1 is linked with a higher metabolic risk in older bariatric patients.
Li et al. [53]	2011	23 patients with T2DM, 22 patients with MetS, 22 patients with T2DM-MetS, 26 healthy people	MCP-1 in the combined T2DM-MetS group was higher in comparison to those in the separate T2DM and MetS groups.
Xu et al. [54]	2015	150 patients with T2DM; 50 healthy patients	A correlation between the MCP-1 A-2518G polymorphism and macrovascular complications in patients suffering from DM.
Oh et al. [55]	2013	31 women with MetS (TLM group), 21 healthy women	The MCP-1 amount rose at a slower pace in the TLM group in comparison to the control group.
Troseid et al. [56]	2004	21 patients with T2DM; 6 healthy people; 9 people in the exercise group; 9 people in the pravastatin group; 10 patients in the combination of pravastatin and exercise.	In the combined exercise groups, there was a reduction in MCP-1 compared to the combined non-exercise groups.

Abbreviation: MCP-1—monocyte chemoattractant protein 1, T2DM-MetS—type 2 diabetic mellitus—metabolic syndrome, TLM—therapeutic lifestyle modification, MetS—metabolic syndrome, BMI—body mass index.

**Table 10:** Summary of studies on the role of MCP-3 (CCL-7) in the skin and skin diseases.

Author	Year	Population	Key Observation
**The Role of MCP-3 (CCL-7) in the Skin and Skin Diseases.**			
Leijs et al. [5]	2021	25 workers exposed to PCB	An elevated level of CCL7, which is a pro-inflammatory chemokine, in the skin of people who were exposed to PCB.
Brunner et al. [58]	2017	59 patients with AD; 22 patients with PsV; 18 healthy patients;	CCL-7 is also an atherosclerosis factor, which is correlated with SCORAD and BSA.
He et al. [59]	2020	71 patients with AD; 37 healthy people;	Pensioners with AD (>60 years old) presented striking upregulation of CCL-7 in comparison to younger people
Yanaba et al. [60]	2006	69 patients with SSc; 28 healthy patients;	Serum levels of CCL-7 are higher in patients dealing with SSc in comparison to healthy people.
Brunner et al. [61]	2015	C57BL/6 (wild-type) mice; Patients with PsV; Healthy patients.	MCP-3 functions as an activator of TNF-α-dependent Th1/Th17-mediated inflammation in psoriatic skin.
Barbarroja et al. [62]	2023	13 patients with PsA, 4 patients with OA	MCP-3 is higher in the synovial fluid of patients with active PsA in comparison to the synovial fluid of patients suffering from OA.

Abbreviation: PCB—polychlorinated biphenyls, CCL7—chemokine (C-C motif) ligand 7, MCP-3—monocyte chemotactic protein—3, BSA—body surface area, SSc—systemic sclerosis, PsA—psoriatic arthritis, PsV—psoriasis vulgaris, Th1—t helper 1 lymphocytes, Th17—t helper 17 lymphocytes, OA—osteoarthritis.

**Table 11:** Summary of the studies on the role of MCP-3 (CCL7) in the metabolic syndrome.

Author	Year	Population	Key Observation
**The Role of MCP-3 (CCL7) in the Metabolic Syndrome.**			
Bradley et al. [63]	1999	Mice NOD/SCID	MCP-3 is linked with Th1, but not Th2 responses, in pancreatic infiltrates.
Jiao et al. [64]	2009	DIO mice, C57BL/6J mice	MCP-3 rises just after only 1 day of a high-fat diet
Lan et al. [65]	2022	33 women with PIH; 33 controls	Logistic regression of MCP-3 serum is negatively correlated with the onset of PIH.

Abbreviation: NOD/SCID—nonobese diabetic/severe combined immunodeficiency, PIH—pregnancy-induced hypertension, DIO—diet-induced obesity.

**Table 12:** Summary of studies on the role of SDF-1α (CXCL12) in the skin, skin diseases, and metabolic syndrome.

Author	Year	Population	Key Observation
**The Role of SDF-1α (CXCL12) in the Skin, Skin Diseases, and Metabolic Syndrome.**			
Zheng et al. [66]	2022	Hair organ culture	CXCL12 is expressed in DFs, and its quantity is elevated in the telogen and catagen stages of the hair cycle.
Zheng et al. [66]	2022	Hair organ culture	Loss of hair is activated and induced by recombinant CXCL12 therapy, which is due to the telogen-to-anagen transformation and the depletion of hair length.
Guo et al. [67]	2019	Adult male Sprague–Dawley rats	SDF-1/CXCR4 plays a key role in the migration of epidermal stem cells and the repair of wounds.
Cao et al. [68]	2016	Human skin samples were obtained from an iridium radiation accident victim.	Expression of SDF-1α is increased in irradiated human skin, in comparison to nonirradiated equivalents.
Zhang et al. [69]	2016	Placenta tissues (nine fetuses: six male and three female).	Excessive expression of SDF-1α might induce chemotaxis of EPCs towards local wounds.
Luo et al. [70]	2022	Rats (250–300 g) of the Sprague–Dawley (SD) species	modRNA transporting SDF-1α accelerates wound healing in the small skin lesions.
Sun et al. [71]	2021	53 patients with AD, 20 healthy patients.	CXCL12 is highly expressed in atopic skin.
Bernat-Peguera et al. [72]	2019	6-week-old male nude mice	Boosting SDF-1/CXCR4 signaling may enhance tumor cell invasion and tumor metastasis.
Skrzeczynska-Moncznik et al. [73]	2009	46 PsV patients, 28 AD patients; 42 healthy individuals;	The homeostatic chemokine CXCL12, along with CMKLR1 cooperation, may modulate pDC influx into psoriatic skin.
Abdelaal et al. [74]	2020	10 patients with PsV, 10 PsA patients with PsA, 20 controls.	Patients dealing with PsA are characterized by more expression of CXCL12 than patients suffering from PsV before treatment, but not after treatment with MTX.

Abbreviation: DFs—dermal fibroblasts, CXCL12—C-X-C motif chemokine 12, modRNA—modified mRNA, MTX—methotrexate, CMKLR—chemokine-like receptor 1, pDC—plasmacytoid dendritic cells.

**Table 13:** Summary of studies on SDF-1α (CXCL12) in metabolic syndrome.

Author	Year	Population	Key Observation
**The role of SDF-1α (CXCL12) in the Metabolic Syndrome.**			
Jung et al. [75]	2009	79 male Caucasian adolescents. 38 (48%) of them had a WC above the 90th percentile.	SDF-1 can be a new indicator for the diagnostic management of obesity-related diseases.
Li et al. [76]	2012	Patients with hyperlipidemia	SDF-1α may function as a marker of hyperlipidemia.
Kim et al. [77]	2014	C57BL/6J male mice	CXCL12 is a required agent for obesity-induced adipose tissue inflammation and systemic insulin resistance.
Liu et al. [78]	2018	350 HTN patients, 483 controls	Different alleles of the *CXCL12* gene impact the risk of hypertension in the Chinese Han population.
Modanwal et al. [79]	2023	Obese and cancer patients	*CXCL12* gene expression is directly proportional to a higher degree of stress and takes part in obesity and cancer progression.
Aboumrad et al. [80]	2007	NOD Thy-1,2 mice	CXCL-12/CXCR4 pathway may have a protective influence on the development of autoimmune diabetes.

Abbreviation: NOD—non-obese diabetic, HTN—hypertension, WC—waist circumference.

**Table 14:** Summary of the studies on the role of RANTES (CCL5) in the skin, skin diseases, particularly in psoriasis.

Author	Year	Population	Key Observation
**The Role of RANTES (CCL5) in the Skin, Skin Diseases.**			
Wakugawa et al. [81]	2001	human keratinocytes	RANTES takes part in skin inflammation
Matsui et al. [82]	2007	Female specific-pathogen-free BALB/c mice	PEG may stimulate infiltration of eosinophils in the skin via RANTES output by epidermal LCs.
Distler et al. [83]	1999	18 patients with SSc	RANTES exists in the early- and long-lasting epidermal cells of human sclerodermatous skin.
Bornscheuer et al. [84]	1999	17 patients with BP, 3 patients with PsV, 3 patients with pemphigus vegetans, 2 pemphigus foliaceus, 2 patients with DH, 2 patients with LAD;	RANTES is not in charge of eosinophilic influx in autoimmune bullous skin diseases
Yamada et al. [85]	1996	10 patients with AD, 5 healthy patients.	RANTES takes part in eosinophil influx and T cell surge in dermal and colonic tissue in patients suffering from AD.
Kato et al. [86]	2006	12 patients with AD, 5 healthy patients	RANTES with receptors CCR5 and CCR3 take part in the influx of eosinophils during chronic inflammation in AD.
Gambichler et al. [87]	2011	20 patients with morphea, 18 healthy people;	Elevation of median chemokine ligand 5/RANTES (CCL5/RANTES) expression is noted in morphea, in comparison to healthy controls.
Puxeddu et al. [88]	2013	87 patients with CSU, 61 healthy people.	RANTES level is elevated in a large number of patients with CSU.
Chong et al. [89]	2015	17 patients with DLE, 12 normal controls	Several M1 macrophage-associated genes, for instance, CCL5, intensified mRNA levels in DLE skin.
Raychaudhuri et al. [29]	1999	8 patients with PsV, 5 biopsies each from non-lesional psoriatic skin, lichen planus, eczematous dermatitis (3 numular eczema, 2 contact dermatitis) skin from healthy controls	Psoriatic keratinocytes are characterized by an elevated level of RANTES in comparison to healthy keratinocytes.
Fukuoka et al. [90]	1998	Neonatal donors	RANTES is located in the intercellular spaces between epidermal keratinocytes, in psoriatic plaques, contrary to the non-lesional and healthy skin.
Johansen et al. [91]	2017	16 psoriatic patients, 6 atopic patients, 6 healthy patients	RANTES expression is higher in psoriatic lesional skin in comparison to nonlesional psoriatic skin.
Kono et al. [92]	2014	17 psoriatic patients, 17 patients with AD	The number of CCL5+ capillary veins is correlated with the maximum rete ridge length and microabscesses in psoriatic skin.
Rateb et al. [93]	2012	25 psoriatic patients	NB-UVB decreases the expression of RANTES mRNA in patients suffering from psoriatic lesions.
Joshi et al. [94]	2019	40 patients with PsV, 25 healthy patients.	Levels of RANTES are decreased in patients suffering from PsV in comparison to healthy people.

Abbreviation: RANTES—regulated on activation, normal T cell expressed and secreted; CCL5—chemokine (C-C motif) ligand 5, CSU—chronic spontaneous urticaria, DLE—discoid lupus erythematosus, DH—dermatitis herpetiformis, LAD—linear IgA dermatosis, BP—Bullous pemphigoid, PsV—psoriasis vulgaris, SSc—systemic sclerosis, LCs—Langerhans cells, AD—atopic dermatitis.

**Table 15:** Summary of studies on RANTES (CCL5) in the metabolic syndrome.

Author	Year	Population	Key Observation
**The Role of RANTES (CCL5) in the Metabolic Syndrome.**			
Ueba et al. [95]	2014	210 healthy Japanese people	Plasma RANTES quantity is linked with MetS.
Gurkan et al. [96]	2016	20 MetS patients with MSG, 20 MetS patients with MSH, 20 systemically healthy subjects with gingivitis, 20 healthy people.	Raised quantities of RANTES in GCF, in MetS patients with gingivitis, can be linked with the presence of increased gingival inflammation by MetS.
Herder et al. [97]	2006	257 participants in a control group; 265 in a lifestyle group.	In the intervention group, the progression to type 2 diabetes was elevated in patients who had an increase in RANTES.
Herrera-May et al. [98]	2020	1325 Mexican mestizos, 625 patients with ACS, 700 healthy controls.	There is a link between CCL5-403 G/A and CCL5-109 G/A polymorphisms with the risk of developing ACS.
Dworacka et al. [99]	2014	138 patients with T2DM, 30 non-diabetic controls.	Elevated RANTES serum levels in patients dealing with type 2 diabetic patients are closely related to postprandial hyperglycemia.
Tokarz et al. [100]	2019	61 diabetic patients (among whom 35 had DR); 25 healthy people	There is an association between RANTES and nonproliferative DR.
Zhang et al. [101]	2014	90 BALB/c mice	An elevated quantity of CCL5 expression may boost DLBCL in mice suffering from T2DM.

Abbreviation: GCF—gingival crevicular fluid, ACS—acute coronary syndrome, DR—diabetic retinopathy, DLBCL—diffuse large B-cell lymphoma.

## Data Availability

No new data were created or analyzed in this study.

## References

[1] Parisi R., Iskandar I.Y.K., Kontopantelis E., Augustin M., Griffiths C.E.M., Ashcroft D.M. (2020). National, regional, and worldwide epidemiology of psoriasis: Systematic analysis and modelling study. BMJ.

[2] Li X., Yang Q., Zheng J., Gu H., Chen K., Jin H., He C., Xu A., Xu J., Zhang J. (2020). Efficacy and safety of a topical moisturizer containing linoleic acid and ceramide for mild-to-moderate psoriasis vulgaris: A multicenter randomized controlled trial. Dermatol. Ther..

[3] Hughes C.E., Nibbs R.J.B. (2018). A guide to chemokines and their receptors. FEBS J..

[4] Phan K., Lee G., Fischer G. (2020). Pediatric psoriasis and association with cardiovascular and metabolic comorbidities: Systematic review and meta-analysis. Pediatr. Dermatol..

[5] Maradit-Kremers H., Dierkhising R.A., Crowson C.S., Icen M., Ernste F.C., McEvoy M.T. (2013). Risk and predictors of cardiovascular disease in psoriasis: A population-based study. Int. J. Dermatol..

[6] Pinter A., Schwarz P., Gerdes S., Simon J.C., Saalbach A., Rush J., Melzer N., Kramps T., Häberle B., Reinhardt M. (2021). Biologic Treatment in Combination with Lifestyle Intervention in Moderate to Severe Plaque Psoriasis and Concomitant Metabolic Syndrome: Rationale and Methodology of the METABOLyx Randomized Controlled Clinical Trial. Nutrients.

[7] Zdanowska N., Kasprowicz-Furmańczyk M., Placek W., Owczarczyk-Saczonek A. (2021). The Role of Chemokines in Psoriasis—An Overview. Medicina.

[8] Fratton Z., Maione V., Bighetti S., Bettolini L., Stinco G., Errichetti E. (2025). Real-life Experience of Bimekizumab in 27 Obese Patients with Plaque-type Psoriasis: A 24-week Multicenter Retrospective Study. Dermatol. Pract. Concept..

[9] Fratton Z., Bighetti S., Bettolini L., Maione V., Arisi M., Buligan C., Stinco G., Errichetti E. (2025). Real-World Experience of Bimekizumab in a Cohort of 109 Patients over 48 Weeks and Identification of Predictive Factors for an Early Super Response and Risk of Adverse Events. Psoriasis Targets Ther..

[10] Ikumi K., Odanaka M., Shime H., Imai M., Osaga S., Taguchi O., Nishida E., Hemmi H., Kaisho T., Morita A. (2019). Hyperglycemia Is Associated with Psoriatic Inflammation in Both Humans and Mice. J. Investig. Dermatol..

[11] Dulkys Y., Schramm G., Kimmig D., Knöss S., Weyergraf A., Kapp A., Elsner J. (2001). Detection of mRNA for eotaxin-2 and eotaxin-3 in human dermal fibroblasts and their distinct activation profile on human eosinophils. J. Investig. Dermatol..

[12] Owczarek W., Paplińska M., Targowski T., Jahnz-Rózyk K., Paluchowska E., Kucharczyk A., Kasztalewicz B. (2010). Analysis of eotaxin 1/CCL11, eotaxin 2/CCL24 and eotaxin 3/CCL26 expression in lesional and non-lesional skin of patients with atopic dermatitis. Cytokine.

[13] Yawalkar N., Uguccioni M., Schärer J., Braunwalder J., Karlen S., Dewald B., Braathen L.R., Baggiolini M. (1999). Enhanced expression of eotaxin and CCR3 in atopic dermatitis. J. Investig. Dermatol..

[14] Hossny E., Aboul-Magd M., Bakr S. (2001). Increased plasma eotaxin in atopic dermatitis and acute urticaria in infants and children. Allergy.

[15] Park C.W., Lee B.H., Han H.J., Lee C.H., Ahn H.K. (2005). Tacrolimus decreases the expression of eotaxin, CCR3, RANTES and interleukin-5 in atopic dermatitis. Br. J. Dermatol..

[16] Frezzolini A., Teofoli P., Cianchini G., Barduagni S., Ruffelli M., Ferranti G., Puddu P., De Pita O. (2002). Increased expression of eotaxin and its specific receptor CCR3 in bullous pemphigoid. Eur. J. Dermatol..

[17] Günther C., Wozel G., Meurer M., Pfeiffer C. (2011). Up-regulation of CCL11 and CCL26 is associated with activated eosinophils in bullous pemphigoid. Clin. Exp. Immunol..

[18] Bock O., Kreiselmeyer I., Mrowietz U. (2001). Expression of dipeptidyl-peptidase IV (CD26) on CD8+ T cells is significantly decreased in patients with psoriasis vulgaris and atopic dermatitis. Exp. Dermatol..

[19] Vasudevan A.R., Wu H., Xydakis A.M., Jones P.H., Smith E.O., Sweeney J.F., Corry D.B., Ballantyne C.M. (2006). Eotaxin and obesity. J. Clin. Endocrinol. Metab..

[20] Herder C., Haastert B., Müller-Scholze S., Koenig W., Thorand B., Holle R., Wichmann H.E., Scherbaum W.A., Martin S., Kolb H. (2005). Association of systemic chemokine concentrations with impaired glucose tolerance and type 2 diabetes: Results from the Cooperative Health Research in the Region of Augsburg Survey S4 (KORA S4). Diabetes.

[21] Falcone C., Buzzi M.P., Bozzini S., Boiocchi C., D’Angelo A., Schirinzi S., Choi J., Kilama M.O., Esposito C., Torreggiani M. (2013). TALENT Investigators. Relationship between sRAGE and eotaxin-3 with CRP in hypertensive patients at high cardiovascular risk. J. Nephrol..

[22] Loughrey B.V., McGinty A., Young I.S., McCance D.R., Powell L.A. (2013). Increased circulating CC chemokine levels in the metabolic syndrome are reduced by low-dose atorvastatin treatment: Evidence from a randomized controlled trial. Clin. Endocrinol. (Oxf.).

[23] Li J., Thornhill M.H. (2000). Growth-regulated peptide-alpha (GRO-alpha) production by oral keratinocytes: A comparison with skin keratinocytes. Cytokine.

[24] Schaper-Gerhardt K., Hansel A., Walter A., Grimmelmann I., Gutzmer R. (2021). Sirolimus diminishes the expression of GRO-α (CXCL-1) /CXCR2 axis in human keratinocytes and cutaneous squamous cell carcinoma cells. J. Dermatol. Sci..

[25] Kojima T., Cromie M.A., Fisher G.J., Voorhees J.J., Elder J.T. (1993). GRO-alpha mRNA is selectively overexpressed in psoriatic epidermis and is reduced by cyclosporin A in vivo, but not in cultured keratinocytes. J. Investig. Dermatol..

[26] Gillitzer R., Ritter U., Spandau U., Goebeler M., Bröcker E.B. (1996). Differential expression of GRO-alpha and IL-8 mRNA in psoriasis: A model for neutrophil migration and accumulation In Vivo. J. Investig. Dermatol..

[27] König A., Krenn V., Toksoy A., Gerhard N., Gillitzer R. (2000). Mig, GRO alpha and RANTES messenger RNA expression in lining layer, infiltrates and different leucocyte populations of synovial tissue from patients with rheumatoid arthritis, psoriatic arthritis and osteoarthritis. Virchows Arch..

[28] Kulke R., Bornscheuer E., Schlüter C., Bartels J., Röwert J., Sticherling M., Christophers E. (1998). The CXC receptor 2 is overexpressed in psoriatic epidermis. J. Investig. Dermatol..

[29] Raychaudhuri S.P., Jiang W.Y., Farber E.M., Schall T.J., Ruff M.R., Pert C.B. (1999). Upregulation of RANTES in psoriatic keratinocytes: A possible pathogenic mechanism for psoriasis. Acta Derm. Venereol..

[30] Zeng J., Chen X., Lei K., Wang D., Lin L., Wang Y., Li Y., Liu Y., Zhang L., Zuo D. (2019). Mannan-binding lectin promotes keratinocyte to produce CXCL1 and enhances neutrophil infiltration at the early stages of psoriasis. Exp. Dermatol..

[31] Kato Y., Yamamoto T. (2010). Increased serum levels of growth-related oncogene-alpha in patients with generalized pustular psoriasis. Dermatology.

[32] Takahashi K., Ohara M., Sasai T., Homma H., Nagasawa K., Takahashi T., Yamashina M., Ishii M., Fujiwara F., Kajiwara T. (2011). Serum CXCL1 concentrations are elevated in type 1 diabetes mellitus, possibly reflecting activity of anti-islet autoimmune activity. Diabetes Metab. Res. Rev..

[33] Feng S.T., Yang Y., Yang J.F., Gao Y.M., Cao J.Y., Li Z.L., Tang T.T., Lv L.L., Wang B., Wen Y. (2021). Urinary sediment CCL5 messenger RNA as a potential prognostic biomarker of diabetic nephropathy. Clin. Kidney J..

[34] Darakhshan S., Fatehi A., Hassanshahi G., Mahmoodi S., Hashemi M.S., Karimabad M.N. (2019). Serum concentration of angiogenic (CXCL1, CXCL12) and angiostasis (CXCL9, CXCL10) CXC chemokines are differentially altered in normal and gestational diabetes mellitus associated pregnancies. J. Diabetes Metab. Disord..

[35] Sajadi S.M.A., Khoramdelazad H., Hassanshahi G., Rafatpanah H., Hosseini J., Mahmoodi M., Arababadi M.K., Derakhshan R., Hasheminasabzavareh R., Hosseini-Zijoud S.M. (2013). Plasma levels of CXCL1 (GRO-alpha) and CXCL10 (IP-10) are elevated in type 2 diabetic patients: Evidence for the involvement of inflammation and angiogenesis/angiostasis in this disease state. Clin. Lab..

[36] Nunemaker C.S., Chung H.G., Verrilli G.M., Corbin K.L., Upadhye A., Sharma P.R. (2014). Increased serum CXCL1 and CXCL5 are linked to obesity, hyperglycemia, and impaired islet function. J. Endocrinol..

[37] Johny E., Bhaskar P., Alam M.J., Kuladhipati I., Das R., Adela R. (2021). Platelet Mediated Inflammation in Coronary Artery Disease with Type 2 Diabetes Patients. J. Inflamm. Res..

[38] Tang J., Lee C.A., Du Y., Sun Y., Pearlman E., Sheibani N., Kern T.S. (2013). MyD88-dependent pathways in leukocytes affect the retina in diabetes. PLoS ONE.

[39] Wang L., Zhang Y.L., Lin Q.Y., Liu Y., Guan X.M., Ma X.L., Cao H.J., Liu Y., Bai J., Xia Y.L. (2018). CXCL1-CXCR2 axis mediates angiotensin II-induced cardiac hypertrophy and remodelling through regulation of monocyte infiltration. Eur. Heart J..

[40] Camnitz W., Burdick M.D., Strieter R.M., Mehrad B., Keeley E.C. (2012). Dose-dependent Effect of Statin Therapy on Circulating CXCL12 Levels in Patients with Hyperlipidemia. Clin. Transl. Med..

[41] Akhtar S., Hartmann P., Karshovska E., Rinderknecht F.A., Subramanian P., Gremse F., Grommes J., Jacobs M., Kiessling F., Weber C. (2015). Endothelial Hypoxia-Inducible Factor-1α Promotes Atherosclerosis and Monocyte Recruitment by Upregulating MicroRNA-19a. Hypertension.

[42] Yamashiro S., Takeya M., Kuratsu J., Ushio Y., Takahashi K., Yoshimura T. (1998). Intradermal injection of monocyte chemoattractant protein-1 induces emigration and differentiation of blood monocytes in rat skin. Int. Arch. Allergy Immunol..

[43] Kunstfeld R., Lechleitner S., Wolff K., Petzelbauer P. (1998). MCP-1 and MIP-1alpha are most efficient in recruiting T cells into the skin In Vivo. J. Investig. Dermatol..

[44] Nakamura K., Williams I.R., Kupper T.S. (1995). Keratinocyte-derived monocyte chemoattractant protein 1 (MCP-1): Analysis in a transgenic model demonstrates MCP-1 can recruit dendritic and Langerhans cells to skin. J. Investig. Dermatol..

[45] Shallo H., Plackett T.P., Heinrich S.A., Kovacs E.J. (2003). Monocyte chemoattractant protein-1 (MCP-1) and macrophage infiltration into the skin after burn injury in aged mice. Burns.

[46] Tabatabaei-Panah P.S., Moravvej H., Hajihasani M., Mousavi M., Ludwig R.L., Akbarzadeh R. (2022). The MCP-1 rs1024611 and MTHFR rs1801133 gene variations and expressions in alopecia areata: A pilot study. Immun. Inflamm. Dis..

[47] Mehta N.N., Li K., Szapary P., Krueger J., Brodmerkel C. (2013). Modulation of cardiometabolic pathways in skin and serum from patients with psoriasis. J. Transl. Med..

[48] Giustizieri M.L., Mascia F., Frezzolini A., De Pità O., Chinni L.M., Giannetti A., Girolomoni G., Pastore S. (2001). Keratinocytes from patients with atopic dermatitis and psoriasis show a distinct chemokine production profile in response to T cell-derived cytokines. J. Allergy Clin. Immunol..

[49] Zablotna M., Sobjanek M., Purzycka-Bohdan D., Szczerkowska-Dobosz A., Nedoszytko B., Nowicki R. (2016). The -2518 A/G MCP-1 and -403 G/A RANTES promoter gene polymorphisms are associated with psoriasis vulgaris. Clin. Exp. Dermatol..

[50] Dai Y.J., Li Y.Y., Zeng H.M., Liang X.A., Xie Z.J., Zheng Z.A., Pan Q.H., Xing Y.X. (2014). Effect of pharmacological intervention on MIP-1α, MIP-1β and MCP-1 expression in patients with psoriasis vulgaris. Asian Pac. J. Trop. Med..

[51] Lembo S., Capasso R., Balato A., Cirillo T., Flora F., Zappia V., Balato N., Ingrosso D., Ayala F. (2014). MCP-1 in psoriatic patients: Effect of biological therapy. J. Dermatol. Treat..

[52] Malin S.K., Kaplan J.L., Meng L., Garmey J.C., Kirby J.L., Taylor A.M., Hallowell P.T., McNamara C.A. (2017). Age increases MCP-1 level in association with bariatric surgery operating time and metabolic risk severity. Obes. Sci. Pract..

[53] Li H., Deng X., Li Z., Luo C., Liu J., Wang Y. (2011). Variation of serum monocyte chemoattractant protein-1 in patients with diabetes and metabolic syndrome. J. Huazhong Univ. Sci. Technol. Med. Sci..

[54] Xu J., Liao Y.F., Zhou W.P., Ming H.L., Wang Q.H. (2015). The MCP-1 Gene A-2518G Polymorphism Confers an Increased Risk of Vascular Complications in Type 2 Diabetes Mellitus Patients. Genet. Test. Mol. Biomark..

[55] Oh E.G., Bang S.Y., Kim S.H., Hyun S.S., Chu S.H., Jeon J.Y., Im J.A., Lee J.E., Lee M.K. (2013). Therapeutic lifestyle modification program reduces plasma levels of the chemokines CRP and MCP-1 in subjects with metabolic syndrome. Biol. Res. Nurs..

[56] Trøseid M., Lappegård K.T., Claudi T., Damås J.K., Mørkrid L., Brendberg R., Mollnes T.E. (2004). Exercise reduces plasma levels of the chemokines MCP-1 and IL-8 in subjects with the metabolic syndrome. Eur. Heart J..

[57] Leijs M., Fietkau K., Merk H.F., Schettgen T., Kraus T., Esser A. (2021). Upregulation of CCL7, CCL20, CXCL2, IL-1β, IL-6 and MMP-9 in Skin Samples of PCB Exposed Individuals-A Preliminary Study. Int. J. Environ. Res. Public Health.

[58] Brunner P.M., Suárez-Fariñas M., He H., Malik K., Wen H.C., Gonzalez J., Chan T.C., Estrada Y., Zheng X., Khattri S. (2017). The atopic dermatitis blood signature is characterized by increases in inflammatory and cardiovascular risk proteins. Sci. Rep..

[59] He H., Li R., Choi S., Zhou L., Pavel A., Estrada Y.D., Krueger J.G., Guttman-Yassky E. (2020). Increased cardiovascular and atherosclerosis markers in blood of older patients with atopic dermatitis. Ann. Allergy Asthma Immunol..

[60] Yanaba K., Komura K., Kodera M., Matsushita T., Hasegawa M., Takehara K., Sato S. (2006). Serum levels of monocyte chemotactic protein-3/CCL7 are raised in patients with systemic sclerosis: Association with extent of skin sclerosis and severity of pulmonary fibrosis. Ann. Rheum. Dis..

[61] Brunner P.M., Glitzner E., Reininger B., Klein I., Stary G., Mildner M., Uhrin P., Sibilia M., Stingl G. (2015). CCL7 contributes to the TNF-alpha-dependent inflammation of lesional psoriatic skin. Exp. Dermatol..

[62] Barbarroja N., López-Montilla M.D., Cuesta-López L., Pérez-Sánchez C., Ruiz-Ponce M., López-Medina C., Ladehesa-Pineda M.L., López-Pedrera C., Escudero-Contreras A., Collantes-Estévez E. (2023). Characterization of the inflammatory proteome of synovial fluid from patients with psoriatic arthritis: Potential treatment targets. Front. Immunol..

[63] Bradley L.M., Asensio V.C., Schioetz L.K., Harbertson J., Krahl T., Patstone G., Woolf N., Campbell I.L., Sarvetnick N. (1999). Islet-specific Th1, but not Th2, cells secrete multiple chemokines and promote rapid induction of autoimmune diabetes. J. Immunol..

[64] Jiao P., Chen Q., Shah S., Du J., Tao B., Tzameli I., Yan W., Xu H. (2009). Obesity-related upregulation of monocyte chemotactic factors in adipocytes: Involvement of nuclear factor-kappaB and c-Jun NH2-terminal kinase pathways. Diabetes.

[65] Lan X., Guo L., Zhu S., Cao Y., Niu Y., Han S., Li Z., Li Y., Yan J. (2022). First-Trimester Serum Cytokine Profile in Pregnancies Conceived After Assisted Reproductive Technology (ART) with Subsequent Pregnancy-Induced Hypertension. Front. Immunol..

[66] Zheng M., Oh S.H., Choi N., Choi Y.J., Kim J., Sung J.H. (2022). CXCL12 inhibits hair growth through CXCR4. Biomed. Pharmacother..

[67] Guo R., Chai L., Chen L., Chen W., Ge L., Li X., Li H., Li S., Cao C. (2015). Stromal cell-derived factor 1 (SDF-1) accelerated skin wound healing by promoting the migration and proliferation of epidermal stem cells. Vitr. Cell. Dev. Biol.-Anim..

[68] Cao J., Zhu W., Yu D., Pan L., Zhong L., Xiao Y., Gao Y., Jiao Y., Zhang Q., Ji J. (2019). The Involvement of SDF-1α/CXCR4 Axis in Radiation-Induced Acute Injury and Fibrosis of Skin. Radiat. Res..

[69] Zhang Y.Q., Ji S.Z., Fang H., Zheng Y.J., Luo P.F., Wu H.B., Wu M.J., Wang Z.H., Xiao S.C., Xia Z.F. (2016). Use of Amniotic Microparticles Coated with Fibroblasts Overexpressing SDF-1α to Create an Environment Conducive to Neovascularization for Repair of Full-Thickness Skin Defects. Cell Transplant..

[70] Luo Z., Bian Y., Zheng R., Song Y., Shi L., Xu H., Wang H., Li X., Tao Z., Wang A. (2022). Combination of chemically modified SDF-1α mRNA and small skin improves wound healing in diabetic rats with full-thickness skin defects. Cell Prolif..

[71] Sun Z.W., Kim J.H., Kim S.H., Kim H.R., Zhang K., Pan Y., Ko M.K., Kim B.M., Chu H., Lee H.R. (2021). Skin-resident natural killer T cells participate in cutaneous allergic inflammation in atopic dermatitis. J. Allergy Clin. Immunol..

[72] Bernat-Peguera A., Simón-Extremera P., da Silva-Diz V., López de Munain M., Díaz-Gil L., Penin R.M., González-Suárez E., Pérez Sidelnikova D., Bermejo O., Viñals J.M. (2019). PDGFR-induced autocrine SDF-1 signaling in cancer cells promotes metastasis in advanced skin carcinoma. Oncogene.

[73] Skrzeczyńska-Moncznik J., Wawro K., Stefańska A., Oleszycka E., Kulig P., Zabel B.A., Sułkowski M., Kapińska-Mrowiecka M., Czubak-Macugowska M., Butcher E.C. (2009). Potential role of chemerin in recruitment of plasmacytoid dendritic cells to diseased ski. Biochem. Biophys. Res. Commun..

[74] Abdelaal N.H., Elhefnawy N.G., Abdulmonem S.R., Sayed S., Saleh N.A., Saleh M.A. (2020). Evaluation of the expression of the stromal cell-derived factor-1 alpha (CXCL 12) in psoriatic patients after treatment with Methotrexate. J. Cosmet. Dermatol..

[75] Jung C., Fischer N., Fritzenwanger M., Pernow J., Brehm B.R., Figulla H.R. (2009). Association of waist circumference, traditional cardiovascular risk factors, and stromal-derived factor-1 in adolescents. Pediatr. Diabetes.

[76] Li S.L., Lin W., Zhang Y., Zheng Z.C., Liu L.J., Fu H., Liu J., Wang G.D., Chen S.Y., Feng L.H. (2012). Stromal cell-derived factor-1α as a novel biomarker for hyperlipidemia. Tohoku J. Exp. Med..

[77] Kim D., Kim J., Yoon J.H., Ghim J., Yea K., Song P., Park S., Lee A., Hong C.P., Jang M.S. (2014). CXCL12 secreted from adipose tissue recruits macrophages and induces insulin resistance in mice. Diabetologia.

[78] Liu X., Chen L., Zhang Y., Wu X., Zhao Y., Wu X., Chen W., Wu C., Chen Y. (2018). Associations between polymorphisms of the CXCL12 and CNNM2 gene and hypertension risk: A case-control study. Gene.

[79] Modanwal S., Mishra N. (2023). Identification of common genes in obesity and cancer through network interaction and targeting those genes by virtual screening approach. J. Biomol. Struct. Dyn..

[80] Aboumrad E., Madec A.M., Thivolet C. (2007). The CXCR4/CXCL12 (SDF-1) signalling pathway protects non-obese diabetic mouse from autoimmune diabetes. Clin. Exp. Immunol..

[81] Wakugawa M., Nakamura K., Akatsuka M., Nakagawa H., Tamaki K. (2001). Interferon-gamma-induced RANTES production by human keratinocytes is enhanced by IL-1beta, TNF-alpha, IL-4 and IL-13 and is inhibited by dexamethasone and tacrolimus. Dermatology.

[82] Matsui K., Wirotesangthong M., Nishikawa A. (2007). Percutaneous application of peptidoglycan from *Staphylococcus aureus* induces eosinophil infiltration in mouse skin. Clin. Exp. Allergy.

[83] Distler O., Rinkes B., Hohenleutner U., Schölmerich J., Landthaler M., Lang B., Gay S., Müller-Ladner U. (1999). Expression of RANTES in biopsies of skin and upper gastrointestinal tract from patients with systemic sclerosis. Rheumatol. Int..

[84] Bornscheuer E., Zillikens D., Schröder J.M., Sticherling M. (1999). Lack of expression of interleukin 8 and RANTES in autoimmune bullous skin diseases. Dermatology.

[85] Yamada H., Izutani R., Chihara J., Yudate T., Matsukura M., Tezuka T. (1996). RANTES mRNA expression in skin and colon of patients with atopic dermatitis. Int. Arch. Allergy Immunol..

[86] Kato Y., Pawankar R., Kimura Y., Kawana S. (2006). Increased expression of RANTES, CCR3 and CCR5 in the lesional skin of patients with atopic eczema. Int. Arch. Allergy Immunol..

[87] Gambichler T., Skrygan M., Labanski A.A., Kolios A.G., Altmeyer P., Kreuter A. (2011). Significantly increased CCL5/RANTES and CCR7 mRNA levels in localized scleroderma. Regul. Pept..

[88] Puxeddu I., Panza F., Pratesi F., Bartaloni D., Casigliani Rabl S., Rocchi V., Del Corso I., Migliorini P. (2013). CCL5/RANTES, sVCAM-1, and sICAM-1 in chronic spontaneous urticaria. Int. Arch. Allergy Immunol..

[89] Chong B.F., Tseng L.C., Hosler G.A., Teske N.M., Zhang S., Karp D.R., Olsen N.J., Mohan C. (2015). A subset of CD163+ macrophages displays mixed polarizations in discoid lupus skin. Arthritis Res. Ther..

[90] Fukuoka M., Ogino Y., Sato H., Ohta T., Komoriya K., Nishioka K., Katayama I. (1998). RANTES expression in psoriatic skin, and regulation of RANTES and IL-8 production in cultured epidermal keratinocytes by active vitamin D3 (tacalcitol). Br. J. Dermatol..

[91] Johansen C., Rittig A.H., Mose M., Bertelsen T., Weimar I., Nielsen J., Andersen T., Rasmussen T.K., Deleuran B., Iversen L. (2017). STAT2 is involved in the pathogenesis of psoriasis by promoting CXCL11 and CCL5 production by keratinocytes. PLoS ONE.

[92] Kono F., Honda T., Aini W., Manabe T., Haga H., Tsuruyama T. (2014). Interferon-γ/CCR5 expression in invariant natural killer T cells and CCL5 expression in capillary veins of dermal papillae correlate with development of psoriasis vulgaris. Br. J. Dermatol..

[93] Rateb A.A., Fawzi M.M.T., Abdel Hay R.M., Mohammed F.N., Amr K.S. (2012). Reduction of RANTES expression in lesional psoriatic skin after narrow band ultraviolet therapy: A possible marker of therapeutic efficacy. Eur. J. Dermatol..

[94] Joshi N., Narang T., Dogra S., Chhabra S. (2019). Circulating levels of chemokines in patients with psoriasis vulgaris and their association with disease severity: A case-control study from North India. Indian J. Dermatol. Venereol. Leprol..

[95] Ueba T., Nomura S., Inami N., Yokoi T., Inoue T. (2014). Elevated RANTES level is associated with metabolic syndrome and correlated with activated platelets associated markers in healthy younger men. Clin. Appl. Thromb./Haemost..

[96] Gürkan A., Eren G., Çetinkalp S., Akçay Y.D., Emingil G., Atilla G. (2016). Monocyte chemotactic protein-1, RANTES and macrophage migration inhibitory factor levels in gingival crevicular fluid of metabolic syndrome patients with gingivitis. Arch. Oral Biol..

[97] Herder C., Peltonen M., Koenig W., Kräft I., Müller-Scholze S., Martin S., Lakka T., Ilanne-Parikka P., Eriksson J.G., Hämäläinen H. (2006). Systemic immune mediators and lifestyle changes in the prevention of type 2 diabetes: Results from the Finnish Diabetes Prevention Study. Diabetes.

[98] Herrera-Maya G., Vargas-Alarcon G., Ramirez-Bello J., Perez-Mendez O., Posadas-Sanchez R., Lopez-Marure R., Granados J., Nieto-Lima B., Fragoso J.M. (2020). Two genetic variants in the promoter region of the CCL5 gene are associated with the risk of acute coronary syndrome and with a lower plasma CCL5 concentration. Immunol. Lett..

[99] Dworacka M., Krzyżagórska E., Iskakova S., Bekmukhambetov Y., Urazayev O., Dworacki G. (2014). Increased circulating RANTES in type 2 diabetes. Eur. Cytokine Netw..

[100] Tokarz A., Konkolewska M., Kuśnierz-Cabala B., Maziarz B., Hanarz P., Żurakowski A., Szuścik I., Stępień E.Ł. (2019). Retinopathy severity correlates with RANTES concentrations and CCR 5-positive microvesicles in diabetes. Folia Medica Cracov..

[101] Zhang J., Luo J., Liu F., Wu D., Zhong Q., Zeng L., Wu Z., Zhao T., Wu L., Hao H. (2014). Diabetes mellitus potentiates diffuse large B-cell lymphoma via high levels of CCL5. Mol. Med. Rep..

